# Biodegradable magnesium alloy WE43 porous scaffolds fabricated by laser powder bed fusion for orthopedic applications: Process optimization, *in vitro* and *in vivo* investigation

**DOI:** 10.1016/j.bioactmat.2022.02.020

**Published:** 2022-02-24

**Authors:** Jinge Liu, Bingchun Liu, Shuyuan Min, Bangzhao Yin, Bo Peng, Zishi Yu, Caimei Wang, Xiaolin Ma, Peng Wen, Yun Tian, Yufeng Zheng

**Affiliations:** a The State Key Laboratory of Tribology, Tsinghua University, Beijing, 100084, China; b Department of Mechanical Engineering, Tsinghua University, Beijing, 100084, China; c Department of Orthopaedics, Peking University Third Hospital, Beijing, 100191, China; d School of Materials Science and Engineering, Peking University, Beijing, 100871, China; e Engineering Research Center of Bone and Joint Precision Medicine, Ministry of Education, Beijing, 100191, China; f Beijing AKEC Medical Co., Ltd., Beijing, 102200, China

**Keywords:** Additive manufacturing, Biodegradable metal, Laser powder bed fusion, Magnesium alloy, Porous scaffold, WE43

## Abstract

Laser powder bed fusion (L-PBF) of magnesium (Mg) alloy porous scaffolds is expected to solve the dual challenges from customized structures and biodegradable functions required for repairing bone defects. However, one of the key technical difficulties lies in the poor L-PBF process performance of Mg, contributed by the high susceptibility to oxidation, vaporization, thermal expansion, and powder attachment etc. This work investigated the influence of L-PBF energy input and scanning strategy on the formation quality of porous scaffolds by using WE43 powder, and characterized the microstructure, mechanical properties, biocompatibility, biodegradation and osteogenic effect of the as-built WE43 porous scaffolds. With the customized energy input and scanning strategy, the relative density of struts reached over 99.5%, and the geometrical error between the designed and the fabricated porosity declined to below 10%. Massive secondary phases including intermetallic precipitates and oxides were observed. The compressive strength (4.37–23.49 MPa) and elastic modulus (154.40–873.02 MPa) were comparable to those of cancellous bone. Good biocompatibility was observed by *in vitro* cell viability and *in vivo* implantation. The biodegradation of as-built porous scaffolds promoted the osteogenic effect, but the structural integrity devastated after 12 h by the immersion tests in Hank's solution and after 4 weeks by the implantation in rabbits' femur, indicating an excessively rapid degradation rate.

## Highlights

• In vitro and in vivo investigations were performed on WE43 porous scaffolds.
• Reliable fusion quality and dimensional accuracy were achieved.
• The compressive strength and Young modulus ranged 4.37–23.49 and 154.40–873.02 MPa.
• Good biocompatibility and improved osteogenic effect were observed.
• The massive secondary phases as well as the enlarged specific surface resulted to a rapid degradation rate.

## Graphical abstract

**Figure:**
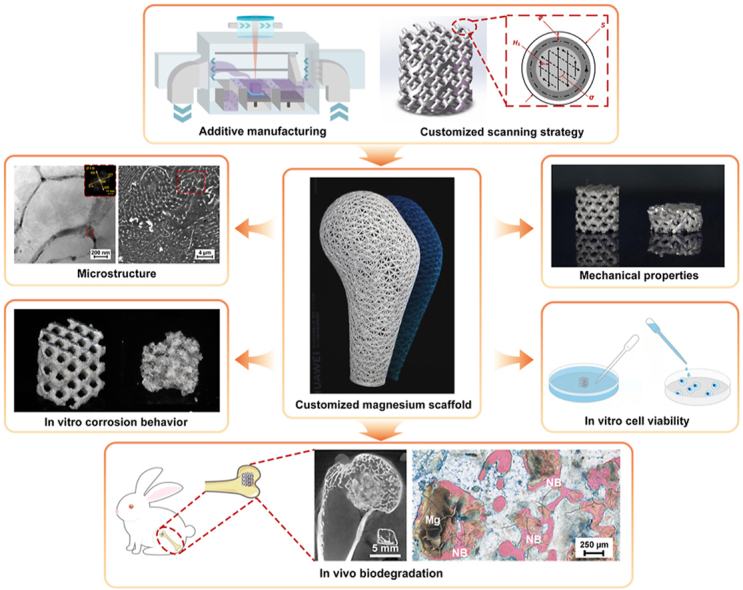

## 1 Introduction

Magnesium (Mg) is the fourth abundant metal element found in human bodies. Mg is bioactive in physiological environment, and involved in more than 300 beneficial enzymatic reactions. The released Mg ion at bone fracture stimulates the formation of calcitonin gene-related peptide (CGRP), and promotes the osteogenic differentiation of stem cells. The density and Young modulus of bulk pure Mg are approximately 1.74 g/cm³ and 40 GPa, close to those of compact cortical bone (1.8 g/cm³ and 30 GPa) [[1], [2], [3], [4]]. With appropriate alloying and fabrication, various biodegradable Mg alloys have been developed with the improved performance. Their orthopedic applications have attracted increasingly attention in the past two decades [[5], [6], [7], [8], [9], [10], [11]].

WE43 alloy was firstly developed for high strength and high temperature applications in 1980s with adding rare earth (RE) elements including Yttrium (Y), Neodymium (Nd) and Gadolinium (Gd). The fine grains and precipitation phases lead to significant strengthening effect. The relatively high solubility, the small difference in electrode potentials, and the passivation effect of RE elements considerably improve the resistance to oxidation and corrosion. WE43 has become one of the very limited biodegradable metals that have achieved clinical applications [12,13]. Biodegradable bone screws and vascular stents based on WE43 alloy passed CE certification respectively in the year of 2013 and 2016 [[14], [15], [16], [17]]. However, they are all regular bulk samples produced by conventional manufacturing processes such as casting and extrusion. Customized porous scaffolds provides major structural merits for bone repairing: patient-specific shapes to fit the geometry of the detected bone and to smoothly bear the load, as well as interconnected internal pores to provide space for ingrowth of bone cells and to avoid the stress shielding effect [18].

Bone has the ability to repair itself, but a large-scale bone defect makes it difficult for natural recovery unless bone grafting is used. Every year, more than two million bone grafting operations are performed all over the world [19]. Additive manufacturing (AM) of porous scaffolds exhibits unrivaled advantages to meet patient-specific needs for the precision treatment of bone defect, and laser powder bed fusion (L-PBF) has been regarded as one of the most appropriate AM methods regarding quality and efficiency [[20], [21], [22]]. Bio-inert metal porous scaffolds fabricated by L-PBF, such as titanium [23] and its alloys [24,25], stainless steels [26], tantalum [27], and cobalt-chromium alloys [28], have been used successfully for the treatment of bone defects. However, the permanent existence of bio-inert metal porous scaffolds obstructs the complete bone tissue reconstruction, and may lead to potential hazards. AM of biodegradable metal porous scaffolds is expected to solve the dual challenges of customized structures and biodegradation, but the L-PBF of Mg alloy porous scaffolds faces great processing challenges resulted by the unique properties of Mg. Firstly, the high reactivity of Mg makes the preparation and operation of Mg alloys powder dangerous. Secondly, Mg has a low boiling point, and the massive vaporization may induce various formation defects. Thirdly, L-PBF of Mg alloys is susceptible to thermal distortion and powder attachment, since Mg has high thermal expansion, good wettability and high surface tension. Finally, hot cracking can be a serious problem for some Mg alloys with low-temperature eutectic reactions [[29], [30], [31]]. The insufficient formation quality has resulted to inconsistent performance, and has put a question mark to the future clinical application of Mg alloy porous scaffolds.

Despite the difficulties, L-PBF of Mg alloys has made great progress in the latest decade [[32], [33], [34], [35], [36], [37], [38], [39], [40], [41], [42], [43], [44], [45], [46], [47], [48], [49], [50], [51], [52], [53], [54]]. Ng et al. firstly attempted L-PBF of pure Mg in 2010 [32]. Jauer et al. achieved L-PBF parts of good formation quality by using AZ91 and WE43 alloys in 2012, and found WE43 had a better processing capability [33]. Wei et al. investigated the effect of vaporization on compositional change by using AZ91 and ZK60 alloys [34,35]. Massive hot cracks were observed during the L-PPBF of ZK60 and JDBM alloys [36,37]. Hot cracking was seldom observed during the L-PBF of WE43 alloy owing to the relatively narrow solidification temperature, however, massive precipitation phases including MgRE compounds and RE oxides were found in WE43 L-PBF parts. Although the strength of WE43 bulks fabricated by L-PBF was higher than casted counterparts and comparable to extruded counterparts, the *in vitro* corrosion rate was higher than those of casted and extruded counterparts [[43], [44], [45], [46], [47], [48], [49]].

Porous scaffolds consist of small-sized struts and interconnected pores enclosed by the struts. In contrast to the widely reported consistent results on the WE43 bulk samples [[43], [44], [45], [46], [47], [48], [49]], limited works investigated WE43 porous scaffolds fabricated by L-PBF [[50], [51], [52], [53]]. The performance of porous scaffolds depends on the structural design and the formation quality, and is sensitive to formation defects and geometrical errors between the design and the fabrication. Formation defects inside the struts reduce loading capacity, and promote pitting corrosion of porous scaffolds. The strength of porous scaffolds decreases exponentially with increasing the structural porosity [[28], [29], [30]]. Most researchers investigated the effect of L-PBF conditions on formation quality by building bulk samples, but the optimized processing conditions for bulk samples may not work for porous scaffolds [54,55]. Y. Li et al. observed 20% loss in volume and 52% loss in strength for WE43 porous scaffolds after 28-day immersion in r-SBF solution [50]. M. Li et al. found that WE43 porous scaffolds exhibited 20% loss in volume and 35% loss in strength after 3-day immersion in DMEM solution, and considerably lost the structural integrity after 7-day immersion [51]. Xie et al. firstly reported the *in vivo* biological behavior and antibacterial properties of Mg alloy porous scaffolds by using Mg-Nd-Zn-Zr powder. However, the fabricated porosity was 32.1%, much lower than the designed value of 80% [56]. Such a big geometrical error definitely causes the uncertainty to performance evaluation, but has been neglected to a great extent in the previous researches. Nevertheless, the biodegradation performance and its effect on biocompatibility and osteogenic effect lack a comprehensive *in vitro* and *in vivo* study.

This work is purposed to develop an optimized L-PBF process to fabricate WE43 porous scaffolds of high fusion quality and dimensional accuracy by using customized energy input and scanning strategy. The microstructure, mechanical properties, biocompatibility, biodegradability and osteogenic effect of the as-built WE43 porous scaffolds are characterized according to *in vitro* and *in vivo* investigations.

## 2 Materials and experimental procedures

### 2.1 Powder characterization

**Fig. 1:**
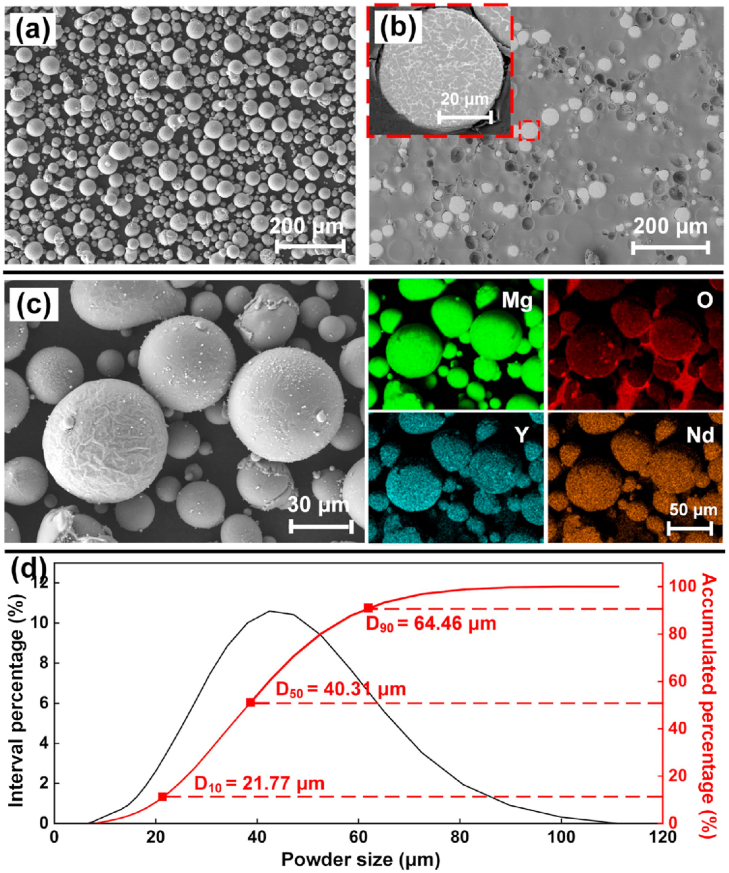
WE43 powder: (a) morphology; (b) cross section; (c) elemental mapping; (d) size distribution.

WE43 cylinder bars were casted in vacuum and then atomized into powder particles by argon gas (Tangshan Weihao, China). Powder particles were dissolved in acid to measure the chemical compositions by inductively coupled plasma optical emission spectroscopy (ICP-OES, iCAP6300) according to GB/T 13748.20–2009. The WE43 powder was composed of 3.87% Y, 2.24% Nd, 1.16% Gd, 0.39% Zr and balance Mg. The content of harmful elements, including Cu, Ni, Cr and Fe, were detected less than 0.001%. As shown in Fig. 1(a), spherical powders with few satellite particles were observed, suggesting good flowability. Fig. 1(b) shows the cross sections of powders, where very few pores were found, indicating little gas inclusion in the powder. Massive precipitation at inter-dendritic regions suggests heavy micro-segregation in the powder, which was contributed to the rapid cooling during atomization. The elemental mapping at powder surface is shown in Fig. 1(c). The main elements of Mg, Y and Nd uniformly distributed from microscopical view, and enriched oxygen was observed at the outmost shells of the powder. The EDS analysis and literature indicated that the oxide shell was majorly composed of Y_2_O_3_, and induced passivation effect, which helped to suppress potential fire accidents [46]. The particle size was measured by a laser diffractometer (BT-9000H, China), and approximately followed a normal distribution as Fig. 1(d) shows. The powder sizes of D10, D50 and D90 in statistics were 21.77, 40.31 and 64.46 μm respectively. The above characterization indicates that the powder quality was suitable to assure a stable L-PBF process.

### 2.2 L-PBF process

**Fig. 2:**
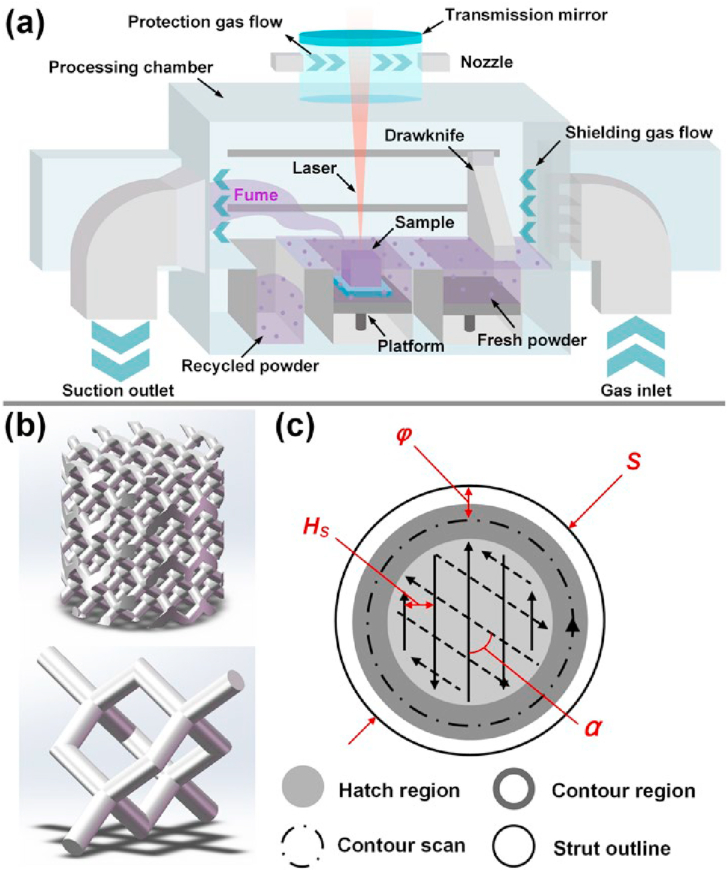
Schematic of L-PBF process (a); the design of a cylindrical porous scaffold of diamond units (b); the scanning strategy at the cross section of the struts (c).

A compact L-PBF machine (BLT S210, China) was used to additively manufacture WE43 porous scaffolds. The optical system consisted of a single mode ytterbium fiber laser (IPG YLR-500, Germany) with the laser spot diameter of 70 μm at the wavelength of 1070 nm. In order to inhibit the negative effect of vaporization during the L-PBF of WE43 powder, a gas circulation system was employed as shown in Fig. 2 (a). Through the enforced argon flow perpendicular to the powder spreading direction, the generated vaporization fume was blown off and suction out from the chamber efficiently. A WE43 rolled plate was used as the substrate in thickness of 20 mm. Before the melting, the WE43 powder bed was preheated to 200 °C. The processing chamber was filled of argon gas with a purity of over 99.99%. When the oxygen content dropped below 80 ppm, the laser was turned on to start melting the powder. During the melting, the oxygen level in the processing chamber was kept below 100 ppm.

As shown in Fig. 2(b), diamond units were adopted to design cylindrical porous scaffolds in outline size of Φ6 × 6 mm^3^. The diameters of struts (*S*) were set as 300, 400 and 500 μm, thus the porous scaffolds were referred as S300D, S400D and S500D which resulted to structural porosity as 89.6%, 82.6%, 72.7% in design, respectively. During L-PBF, the struts were formed by melting the WE43 powder layer upon layer by laser energy input following a designated scanning strategy. Laser power (*P*_*L*_) and scanning speed (*V*_*S*_) were set as variables to investigate the influence of laser energy input on formation quality. Fig. 2(c) shows the illustration of scanning strategy at the cross section of struts. A zig-zag exposure pattern with a 67° rotation (α) each layer was used for the inner hatch region. The hatch spacing (*H*_*S*_) and layer thickness (*t*_*S*_) were fixed as 70 and 20 μm respectively. A contour exposure proportional to the outline of the strut was employed to increase the fabrication accuracy. The same *P*_*L*_ and *V*_*S*_ were used for the hatch and contour region. Offset spacing (*φ*), indicating the distance between the contour line and the strut outline, was set to counteract the size expansion of struts during L-PBF.

### 2.3 Formation quality and microstructure characterization

The formation quality of porous scaffolds was characterized by fusion quality and dimensional accuracy. The fusion quality was evaluated by the relative density of the struts (*ρ*_*S*_). The dimensional accuracy referred to the dimensional error (*Δ*_*P*_) between the designed porosity (*DP*) and the fabricated porosity (*FP*). After the L-PBF process, the fabricated porous scaffolds were put into an ethanol bath to measure the actual volume (*V*_*a*_) by Archimedes method. According to Eq. (1), *M*_*a*_ and *M*_*e*_ were the mass of scaffolds measured in air and ethanol respectively; while *ρ*_*e*_ was the density of ethanol. *Δ*_*P*_ was calculated according to Eq. (2), where *V*_*d*_ was the designed volume of the porous scaffold and was calculated by the 3D data file. Then, the scaffolds were cut perpendicular to the building direction and polished. Five different random regions of struts were captured via an optical microscope (OM, Olympus, Japan). The total area of pores (*A*_*p*_) was measured by employing image processing software, and *ρ*_*S*_ was calculated according to Eq. (3), where *A*_*t*_ was the area of the test region. The final *ρ*_*S*_ was the average value of the five different measurement regions. Compared to Archimedes and CT methods, the image processing method can't provide an overall view of formation defects, however, it works for horizontal comparison and is superior for the delicate observation of formation defects inside the struts.

(1) $$V_{a}=\frac{m_{a}-m_{e}}{\rho_{e}}$$

(2) $$\Delta_{P}=\left(V_{a}-V_{d}\right)/\;V_{d}$$

(3) $$\rho_{S}=1-A_{p}/A_{t}$$

Microstructure was characterized using scanning electron microscopy (SEM, Zeiss Gemini-300) and transmission electron microscopy (TEM, Advantest JEM-2100F). The energy dispersive X-ray spectroscopy (EDS) was applied to analyze chemical compositions of interested regions. Phase identification was carried out by X-ray diffraction (XRD, Bruker D-8) at 40 kV and 200 mA, using a continuous scan mode. A quick scan at 4°/min was conducted over a range of 10–90° to give a general overview of the diffraction peaks.

### 2.4 *In vitro* test of mechanical properties, corrosion behavior and cell viability

The as-built porous scaffolds were compressed at room temperature by a universal tester (Kexin WDW3020, China) at the speed of 0.6 mm/min and with a maximum strain up to 50%. The stress-stain curves were measured to calculate the compressive strength (*CS*) and Young modulus (*YM*). The deformation behavior was captured by a digital camera. Vickers hardness (*VH*) was measured at 10 points at the center of struts with a load of 200 g for 10 s.

Immersion tests were performed to evaluate the corrosion behavior in Hank's solution (37 °C, pH 7.4). Before the immersion, the porous scaffolds were carefully cleaned in pure ethanol by ultrasonic vibration to wipe off the attached powder at the surface. The exposure ratio was set as 20 mL/cm^2^ in accordance with ASTM-G31-72. The released hydrogen volume, pH value and weight loss were sampled per 2 h until the structural integrity of scaffolds was lost. The hydrogen was collected and measured in volume by a narrow-mouthed pycnometer and an Alkali burette. The pH value in the solution was recorded using a pH meter (Sartorius pH PB-10). The weight loss was evaluated by an electronic balance (±1 mg) after removing the corrosion products by CrO_3_ solution. Three replicate samples were measured separately under each condition. After immersion, the samples were cleaned using distilled water, and the corrosion surface was observed by SEM. The corrosion products were further examined by using EDS and XRD.

Cell viability was conducted according to ISO10993−12. Bone mesenchymal stem cells (BMSC) were cultured in the Dulbecco's modified eagle medium (DMEM) with 10% fetal bovine serum, 1% streptomycin and 1% penicillin, in a humidified atmosphere with 5% CO_2_ at 37 °C. The extract was obtained by incubating S400D scaffolds in the same culture medium for 8 h. The extraction ratio was 4 cm^2^/mL, and the supernatant was collected as 100% extract. The Mg ion concentration was 147.4 μg/mL for the 100% extracts. Subsequently, 50% and 10% concentration extracts were produced by mixing various amounts of DMEM. The cytocompatibility assays were evaluated via the 100%, 50%, 10% and 0% (as control) concentration extracts. Cells were incubated in a 96-well culture plate with the density of 3 × 10^3^ cells/well and cultured by DMEM for 24 h to allow cell attachment. Then the medium was replaced with the extracts of different concentrations. After the incubation of 1, 3 and 7 days, the cell viability and proliferation were counted by a Cell Counting Kit-8 (CCK-8, Dojindo, Japan). The spectrophotometric absorbances of each well were measured by a microplate reader (Bio-RAD680) at 450 nm wavelength. For cellular live/dead staining test, cells incubated in different extracts for 1, 3 and 7 days were first rinsed by standard phosphate buffer saline (PBS) and cultured by 2 mM Calcein AM and 4 mM PI (Live/Dead Cell Stains, Dojindo, Japan) for 20 min in a humidified incubator. Then, the cells were rinsed by PBS solution and visualized using a confocal laser scanning microscope (Nikon A1R-si, Japan).

### 2.5 *In vivo* test of biocompatibility, biodegradability and osteogenic effect

Male New Zealand white rabbits 6-month-old with the weight of 3–3.5 kg were enrolled. A lateral condyle cylindrical defect in size of Φ 5 × 6 mm^3^ was built via electric drilling in the left knee of each rabbit. The included 45 rabbits were equally divided into three groups, including untreated group (the defect was left empty), scaffold group (the defect was filled with WE43 porous scaffold), and cement group (the defect was filled with calcium sulfate bone cement). The WE43 and cement samples have approximately the same outline size as that of the defect. At 4, 8 and 12 weeks after the surgery, rabbits were respectively sacrificed by means of euthanasia and their distal femur samples were collected for the following experiment steps. Five samples were harvested at each timing in each group, and timely replacement would be finished once unexpected failure or death occurred. More details on the surgery were described in Fig. S1 in the supplementary materials.

Immediately after the harvest, samples were observed for possible evidences of inflammation, rejection reaction, infection, and fester, which were important indicators to evaluate the *in vivo* biocompatibility of the implants. The rabbits’ venous blood for alanine transaminase (ALT) and UREA detection at 4, 8, and 12 weeks after the surgery was measured to estimate the *in vivo* biotoxicity of WE43 porous scaffolds. The concentration of Mg^2+^ in the blood was also detected. Difference of the ALT, UREA and Mg^2+^ concentration of different groups was compared by the Kruskal-Wallis Test. Statistical analysis was performed by SPSS 20.0 software. *P* < 0.05 was defined as statistically significant difference. The statistical results were displayed by histograms. The vital organs of rabbits sacrificing at each time point were carried out, and the histopathological observation (HE staining) was made to check the expression of brain, heart, liver, spleen, lung and kidney by using optical microscope (Olympus BX53, Japan).

The continuous radiologic evaluations were employed via C-arm fluoroscopy and INVEON Micro-CT scanner (Siemens, Germany). Serial conventional X-ray images presented the conditions of implant stability, degradation, bone regeneration, and related complications. Micro-CT images offered more details on the degradation behavior, the characteristics and process of bone regeneration and reconstruction. Furthermore, the femora were collected and fixed in 10% neutral formalin buffer for 24 h at room temperature. The samples were sliced to 200 μm sections and then were ground to 30–40 μm. Methylene blue/acid fuchsin histological staining was carried out to observe the regeneration of new bone.

## 3 Results

### 3.1 Formation quality

**Fig. 3:**
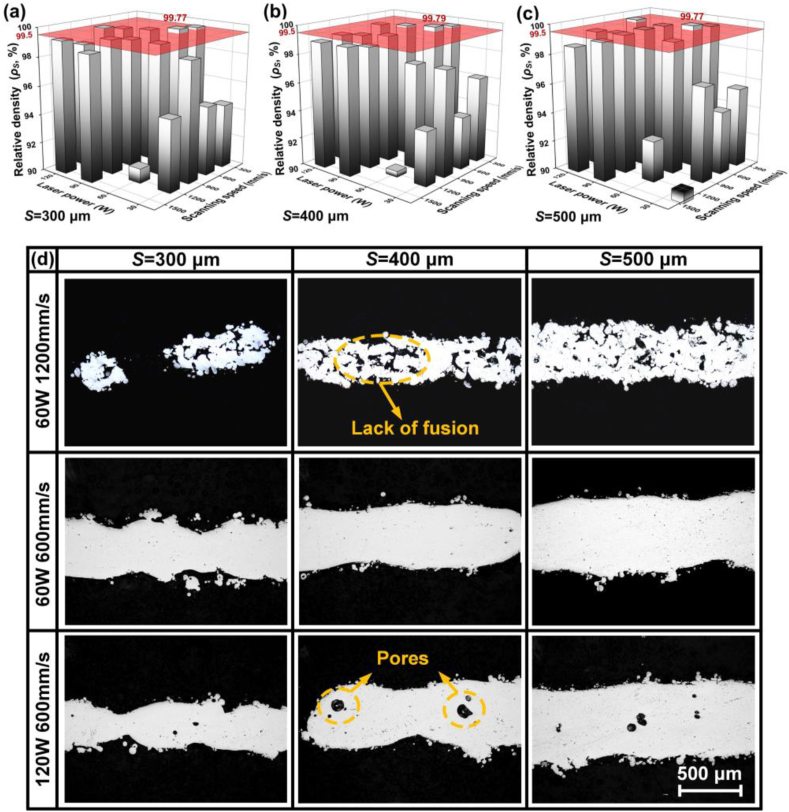
Relative density *ρ*_*S*_ under laser power *P*_*L*_ and scanning speed *V*_*S*_ with strut sizes: (a) 300 μm, (b) 400 μm, (c) 500 μm; (d) typical pictures at cross sections of struts.

Laser power *P*_*L*_ and scanning speed *V*_*S*_ were set as variables, and offset spacing *φ* was fixed as a default value *φ*_*0*_ = 50 μm, to investigate the influence of laser energy input on relative density *ρ*_*S*_ and geometrical error *Δ*_*P*_ during L-PBF of WE43 porous scaffolds. A higher *ρ*_*S*_ and a lower absolute value of *Δ*_*P*_ represents a better fusion quality and a higher dimensional accuracy respectively. As Fig. 3(a–c) shows, *ρ*_*S*_ varied greatly with changing *P*_*L*_ in the range of 30–120 W and *V*_*S*_ of 300–1500 mm/s. *P*_*L*_ and *V*_*S*_ out of the range obviously deteriorated the formation quality, and was out of our research interest. The error bar is not shown in the figure for visual simplicity. The red surface at the top indicates a reliable fusion quality with *ρ*_*S*_ reaching 99.5%. When *P*_*L*_ = 60 W and *V*_*S*_ = 600 mm/s, the maximum *ρ*_*S*_ was obtained as 99.77%, 99.79% and 99.79% for 300, 400 and 500 μm struts respectively. The influence of laser energy input on the fusion quality appeared not affected by the strut size. Fig. 3(d) shows the typical pictures at cross sections of struts. When *P*_*L*_ = 60 W and *V*_*S*_ = 1200 mm/s, namely *P*_*L*_/*V*_*S*_ = 0.5 J/cm, lack of fusion occurred since the energy input was insufficient to completely melt the powder. When *P*_*L*_ = 60 W and *V*_*S*_ = 600 mm/s, namely *P*_*L*_/*V*_*S*_ = 1.0 J/cm, good fusion quality was achieved with few defects. Further increasing *P*_*L*_ to 120 W and *P*_*L*_/*V*_*S*_ to 2.0 J/cm, fusion quality deteriorated with pore defects. With such a high energy input, excessive vaporization resulted to an unstable molten pool, which had a high tendency to entrap the shielding gas to form gas bubbles. The linear heat input *P*_*L*_/*V*_*S*_ (J/cm) represents the energy input per unit length, and a moderate *P*_*L*_/*V*_*S*_ was necessary to assure good fusion quality.

**Fig. 4:**
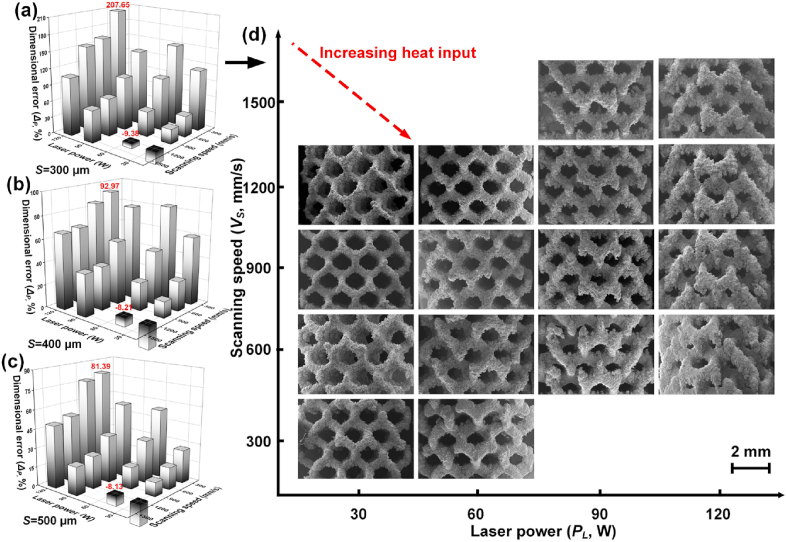
Dimensional error *Δ*_*P*_ under various laser power *P*_*L*_ and scanning speed *V*_*S*_ with sizes: (a) 300 μm; (b) 400 μm; (c) 500 μm, the error bar is not shown for visual simplicity, (d) pictures of S300D porous scaffolds with various laser energy input.

With increasing *P*_*L*_/*V*_*S*_, the width of molten pool increased, and the cooling rate reduced. More surrounding powder particles were wetted and attached to the molten pool. As Fig. 4(a–c) shows, *Δ*_*P*_ varied greatly with changing *P*_*L*_ and *V*_*S*_. The positive *Δ*_*P*_ was mainly explained by the width of molten pool and powder attachment, and was observed under most conditions. Taking S300D porous scaffolds as an example in Fig. 4(d), the biggest *Δ*_*P*_ reached to 207.65% (*P*_*L*_ = 120 W and *V*_*S*_ = 600 mm/s), indicating much thicker struts, smaller pores, and much lower fabricated porosity (*FP*) than the designed values. The negative *Δ*_*P*_ was also observed under limited conditions, which corresponded to insufficient energy input as shown in Fig. 3(a). The insufficient energy input resulted to a small molten pool, less powder attachment and the formation of fusion defects. Thus, thinner struts than the designed values were obtained with the low *ρ*_*S*_ and the negative *Δ*_*P*_. Overall, a small positive *Δ*_*P*_ is required regarding the dimensional accuracy of as-built porous scaffolds. The pictures of S400D and S500D porous scaffolds fabricated by various laser energy input were provided n Figs. S2 and S3 in the supplementary materials.

Although the laser energy input with *P*_*L*_ = 60 W and *V*_*S*_ = 600 mm/s generated the best fusion quality for all the scaffolds, the *Δ*_*P*_ was as high as 96.75%, 47.30% and 33.78%, indicating that the fabricated porosity *FP* was 79.5%, 74.3%, 63.5%, much lower than the designed values respectively for S300D, S400D and S500D scaffolds. Decreasing the laser energy input was beneficial to reduce the *Δ*_*P*_. With the laser energy input of 60 W and 1200 mm/s, the *Δ*_*P*_ even reduced to negative values of −9.38%, −8.21% and −8.13%, however, the *ρ*_*S*_ also reduced to as low as 90.41%, 91.01% and 92.78% respectively for S300D, S400D and S500D scaffolds. A relatively low energy input decreased the dimensional error, but deteriorated the fusion quality; while a relatively high energy input improved the fusion quality, but caused a large positive dimensional error. With the default *φ*, a processing dilemma existed between the pursuit of good fusion quality and high dimensional accuracy, and it was particularly significant for L-PBF of Mg alloys porous scaffolds considering the high susceptibility of Mg to vaporization, powder attachment and thermal expansion.

Besides the laser energy input, the optimization of the offset spacing *φ* was proposed to further improve the formation quality, so called the customized energy input and scanning strategy (CES). In CES, the laser energy input including *P*_*L*_ and *V*_*S*_ was optimized based on the fusion quality, while the *φ*_*c*_ was adjusted according to the geometrical errors measured by using the default *φ*_*0*_ and the optimized *P*_*L*_ and *V*_*S*_. Assuming that the porous scaffolds are made up of a continuous strut with a feature diameter, *S*_*0*_ and *S*_*1*_ are the designed diameter and the fabricated diameter obtained by using *φ*_*0*_; *V*_*0*_ and *V*_*1*_ are the designed volume and the fabricated volume obtained by using *φ*_*0*_; accordingly, the square root of the volume ratio equals to the diameter ratio. Since *V*_*1*_ can be measured by Archimedes method, *φ*_*c*_ can be calculated out by Eq. (4).

(4) $$\varphi_{c}=\frac{S_{1}-S_{0}}{2}=\left(\frac{S_{1}}{S_{0}}-1\right)\frac{S_{0}}{2}=\left(\sqrt{\frac{V_{1}}{V_{0}}}-1\right)\frac{S_{0}}{2}$$

**Fig. 5:**
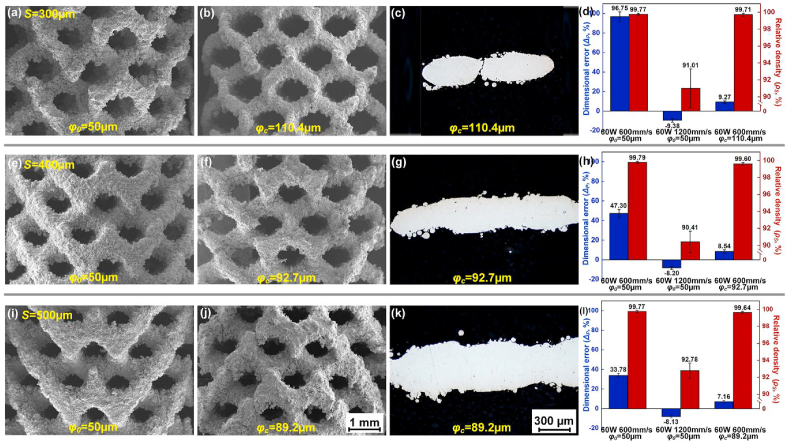
Pictures of surface and cross sections, dimensional error *Δ*_*P*_ and relative density *ρ*_*S*_ of porous scaffolds: (a–d) S300D, (e–h) S400D, (i–l) S500D.

The customized *φ*_*c*_ was dependent upon the strut size in design and the used laser energy input, which thus better compensated the size expansion during the L-PBF. For example, when the laser energy input was set as *P*_*L*_ = 60 W and *V*_*S*_ = 600 mm/s, the *φ*_*c*_ was calculated as 110.4, 92.7 and 89.2 μm respectively for S300D, S400D and S500D scaffolds. Fig. 5 shows the *ρ*_*S*_, *Δ*_*P*_ and the corresponding pictures of porous scaffolds with the default *φ*_*0*_ and the customized *φ*_*c*_ for comparison. With *φ*_*0*_ = 50 μm, either high *ρ*_*S*_ and *Δ*_*P*_ (*P*_*L*_ = 60 W and *V*_*S*_ = 600 mm/s) or low *ρ*_*S*_ and *Δ*_*P*_ (*P*_*L*_ = 60 W and *V*_*S*_ = 1200 mm/s) was obtained. With the customized *φ*_*c*_, high *ρ*_*S*_ greater than 99.5% (99.71%, 99.6% and 99.64%) and low *Δ*_*P*_ smaller than 10% (9.27%, 8.54% and 7.56%) were achieved simultaneously for S300D, S400D and S500D scaffolds.

### 3.2 Microstructure

**Fig. 6:**
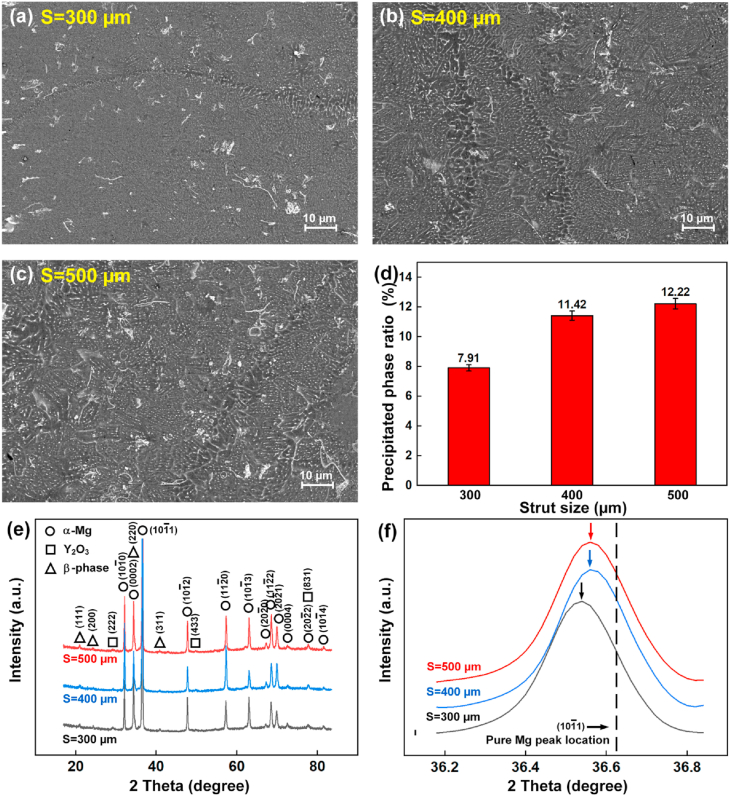
(a–c) Microstructure under SEM observation, (d) precipitated phase ratio, (e–f) XRD pattern of porous scaffolds with different strut sizes.

The as-built WE43 porous scaffolds were composed of 4.26% Y, 2.46% Nd, 1.28% Gd, 0.43% Zr and the residual Mg according to the ICP-OES test. Compared with the starting powder, the concentration of Mg decreased and the concentration of the residual elements increased. The compositional change is explained by the higher vaporization loss of Mg element. Fig. 6(a–c) show the microstructures using the optimized CES conditions. The cross sections were perpendicular to the building direction. Complicated secondary phases in white color were observed including big flakes, particle films along α-Mg grains, and fine dash-line clusters inside α-Mg grains. The ratio of secondary phases at the cross sections increased with increasing the strut sizes. Fig. 6(d) indicates that the ratio of secondary phases in S500D scaffolds was approximately 1.5 times that of S300D scaffolds.

The XRD pattern in Fig. 6(e) implies the presence of α-Mg, Y_2_O_3_ and β phase. The flake phases were speculated as oxides, and were majorly composed of Y_2_O_3_. Oxide shells were observed at the surface of WE43 powder as shown in Fig. 1(c). The melting points of Y_2_O_3_ and MgO are 2410 and 2852 °C; the melting and burning point of pure Mg are 651 and 1107 °C respectively. The oxide shell was crushed into flakes in size of 1–10 μm during the L-PBF, but couldn't be melted since the peak temperature in the molten pool couldn't exceed much more than the boiling point of Mg owing to the vaporization cooling effect. Meanwhile, the formation of Y_2_O_3_ is also possible during the melting, considering that the dissolved Y element in the molten pool can react with the residual oxygen in the L-PBF chamber. β phases are a mixed isomorphous family of Mg_3_X eutectic compounds precipitated from the liquid WE43, where X represents elements Y, Nd, or Gd. They have similar diffraction peaks in the X-ray pattern [[57], [58], [59]].

Compared with the standard pure Mg peak at 36.62°, the diffraction peaks of α-Mg in WE43 scaffolds has an obvious left shift to lower angles as shown in Fig. 6(f). The decreased diffraction angle indicates an increased lattice spacing, which is attributed to the substitutional solid solution of alloying atoms in α-Mg. The atomic radii of the elements (Y: 0.1482 nm; Nd: 0.1773 nm; Gd: 0.1757 nm; Zr: 0.1377 nm) are a bit higher than that of Mg (0.1333 nm) [60,61]. Furthermore, the angles of diffraction peaks decreased with decreasing the strut size (Fig. 6(f)), implying that more alloying atoms were solubilized in α-Mg when a smaller strut size was used.

**Fig. 7:**
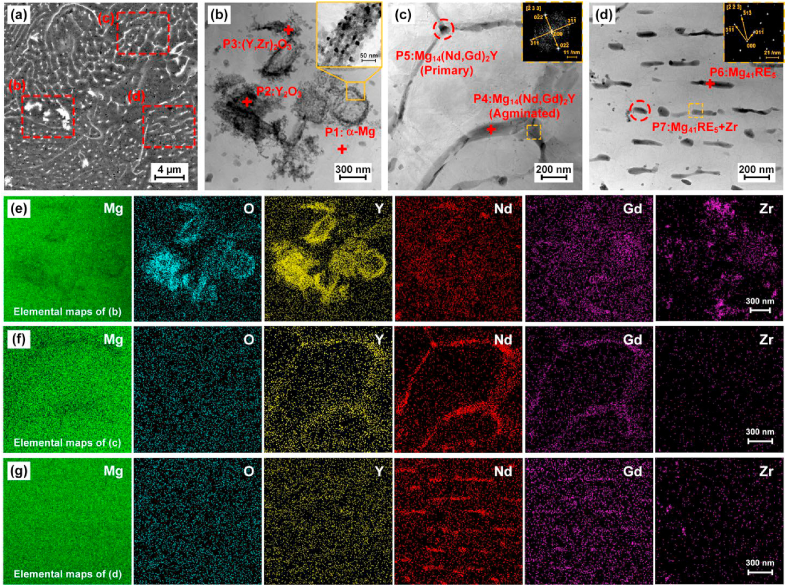
Microstructure under SEM observation (a) and TEM observation (b–d), elemental mapping (e–g) of S500D porous scaffolds.

STEM and its equipped EDS were applied to further characterize the precipitation phases. The samples were cut from S500D scaffolds. The three typical secondary phases are marked in three regions in Fig. 7(a). Their detailed morphology, lattice pattern and elemental mapping are shown in Fig. 7(b–g). The elemental ratios at seven points P1∼P7 are provided in Supplementary Table S1. The enrichment of Y, O and Zr are found at P1 and P2 points, implying the oxides are mainly composed of Y_2_O_3_ and a hybrid oxide of (Y, Zr)_2_O_3_. Little enrichment of Mg, Nd and Gd are observed in oxides. The XRD pattern in Fig. 6(e) and the elemental mapping in Fig. 7(e) both conform this observation. The oxides appear curved flakes in size of 1–10 μm under SEM observation, and are the agglomerates of nano-scale amorphous granules according to the selected area electron diffraction (SAED) in Fig. 7(b).

Region (c) refers to the secondary phases along grain boundaries. The elemental ratios indicates that they are composed of Mg_14_(Nd,Gd)_2_Y, which is a typical type of metastable β phases precipitated in the rapid cooling [58]. Metastable β-Mg_3_Nd, β-Mg_3_Gd and β-Mg_14_Nd_2_Y were widely reported in WE43 casting and L-PBF process [43,44,46,[48], [49], [50], [51], [52]]. Gd replaces Nd in β-Mg_14_Nd_2_Y because of their similar atomic radius. The coexistence and mutual substitution of Y, Nd and Gd were also widely reported in Mg-RE alloys [62]. The enrichment of Y and Nd are clearly observed in Fig. 7(f). The SAED observation in Fig. 7(c) shows that Mg_14_(Nd,Gd)_2_Y has the Fm/3 m group space. The lattice parameter (a = 0.731 nm) of Mg_14_(Nd,Gd)_2_Y approximately matches that of Mg_14_Nd_2_Y reported in literature [57,58]. Region (d) refers to precipitation phases inside the α-Mg substrate. They are compositionally identified as Mg_41_(Nd, Gd)_5_. The enrichment of Nd and Gd is clearly observed in Fig. 7(g). The I4/m group space is detected by the SAED observation with the lattice parameters (a = 1.279 nm; c = 0.904 nm), which approaches to that of Mg_41_Nd_5_ phase in literature [43,63].

### 3.3 Mechanical properties

**Fig. 8:**
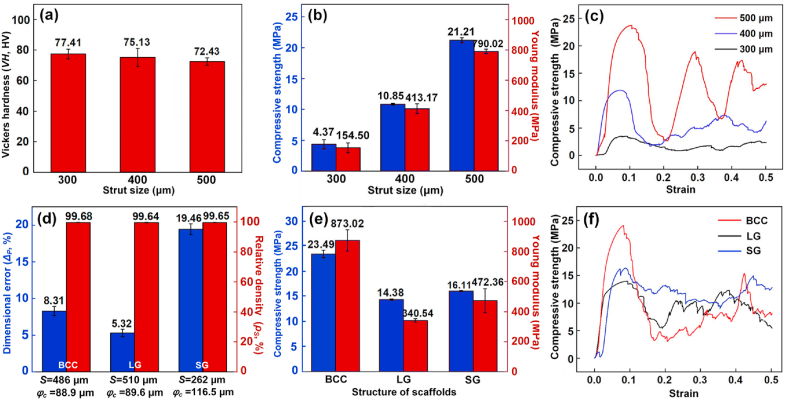
(a) Hardness, (b, e) compressive strength and Young modulus, (c, f) stress-strain curves and (d) dimensional error *Δ*_*P*_ and relative density *ρ*_*S*_ of porous scaffolds with different strut sizes and porous units.

Fig. 8 shows the hardness and compressive properties of the as-built WE43 scaffolds under the optimized CES conditions. The average hardness was approximately 75 HV, and the hardness slightly decreased with increasing the strut size as shown in Fig. 8(a). The hardness of WE43 alloys is hugely influenced by fusion quality and microstructure, and was reported as 50–70 HV and 70–130 HV for casted and extruded samples in literature [64,65]. Compressive strength (*CS*) and Young modulus (*YM*) sharply increased with increasing the strut size, namely the structural porosity, as shown in Fig. 8(b). Compared with S300D scaffolds (*FP* = 88.6%), the *CS* and *YM* of S500D scaffolds (*FP* = 70.8%) increased from 4.37 to 21.21 MPa and from 154.5 to 790.2 MPa respectively. The fluctuation in stress-strain curves in Fig. 8(c) implies a step-by-step crushing, indicating the capacity to bear large deformation. No brittle integral fracture occurred even when 50% compressive strain was applied.

In addition to structural porosity, the mechanical performance of porous scaffolds is also dependent on the shape of porous units as shown in Fig. 8(d–f). The CES was attempted to fabricate porous scaffolds with different types of porous units including body centered cube (BCC, S_0_ = 486 μm), lattice gyroid (LG, S_0_ = 510 μm) and sheet gyroid (SG, S_0_ = 262 μm). Their structural design was shown in Fig. S4 in supplementary materials, and their porosities in design were set roughly as 80%, as the similar as that of S400D scaffolds. Fig. 8(d) shows the *ρ*_*S*_ and *Δ*_*P*_ by using the optimized laser energy input of *P*_*L*_ = 60 W and *V*_*S*_ = 600 mm/s as well as the customized *φ*_*c*_, which was predicted by using the fitted data of S300D, S400D and S500D scaffolds as shown in Fig. S4. All the *ρ*_*S*_ were greater than 99.50%, indicating good fusion quality. For BCC and LG porous scaffolds, the *Δ*_*P*_ was less than 10%, however, the *Δ*_*P*_ was approximately 20% for SG porous scaffolds. The SG scaffolds were made up of sheets rather than struts, and the thickness of sheet was much smaller than the diameter of struts of the other scaffolds. Even with the similar porosity in design, the maximum *CS* and *YM* are roughly 2.3 and 2.6 times the minimum ones among the four different pore units, indicating a great extent to modulate the mechanical performance by adjusting porous unites, which was also reported in literature [66]. The compressive properties of human bone vary with its density and detailed structure. When the bone mineral density is 30%, the *CS* and *YM* of cancellous bone range 0.8–11 MPa and 12–140 MPa [67]. With appropriate porous design and formation quality, WE43 porous scaffolds fabricated by L-PBF provide tremendous advantages to match their mechanical performance to those of different human bones.

### 3.4 *In vitro* corrosion and cell viability

**Fig. 9:**
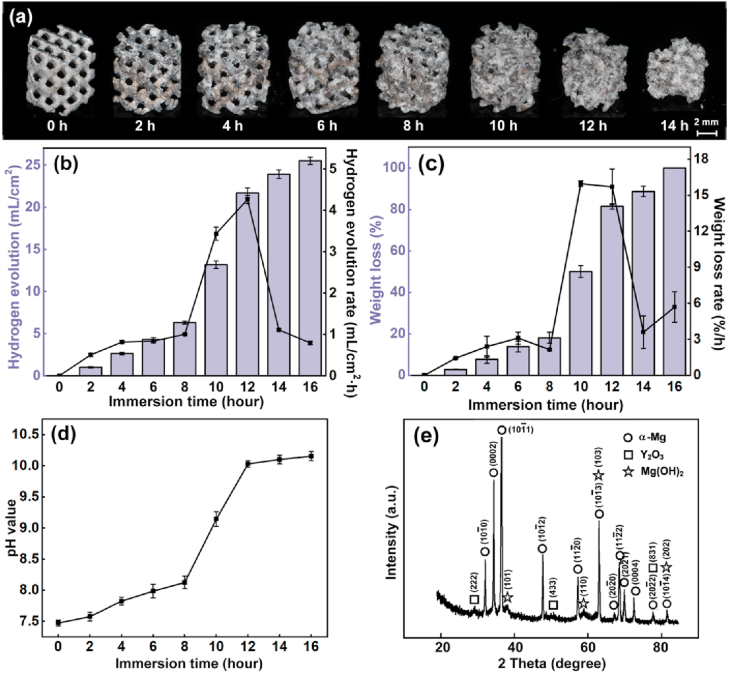
*In vitro* corrosion behavior (a) pictures of porous scaffolds, (b) hydrogen generation, (c) weight loss, (d) pH value after different immersion periods; (e) XRD analysis of corrosion products after 8 h immersion.

S400D porous scaffolds were used for the investigation on *in vitro* corrosion by immersion tests and cell viability by indirect contact tests. Fig. 9(a) shows the pictures of porous scaffolds every 2 h after the immersion in Hank's solution. Only after 4 h, the struts at the corners disappeared. The pores were stuffed with corrosion products after 8 h. The structural integrity lost after 12 h. The hydrogen evolution, pH value and weight loss of the WE43 scaffolds with various immersion periods are shown in Fig. 9(b–d). The hydrogen generation rate was less than 1 mL/cm^2^·h at the initial 8 h, then increased rapidly to the maximum value of 4.26 ml/cm^2^·h, further decreased due to the small residual volume of scaffolds. The hydrogen generation volume, the pH value and the weight loss continuously increased with the immersion time. The weight loss rate followed the change of the hydrogen generation rate. The peak pH value exceeded over 10, the total weight loss reached almost 100%, and the scaffolds collapsed into small pieces after 16 h immersion eventually.

**Fig. 10:**
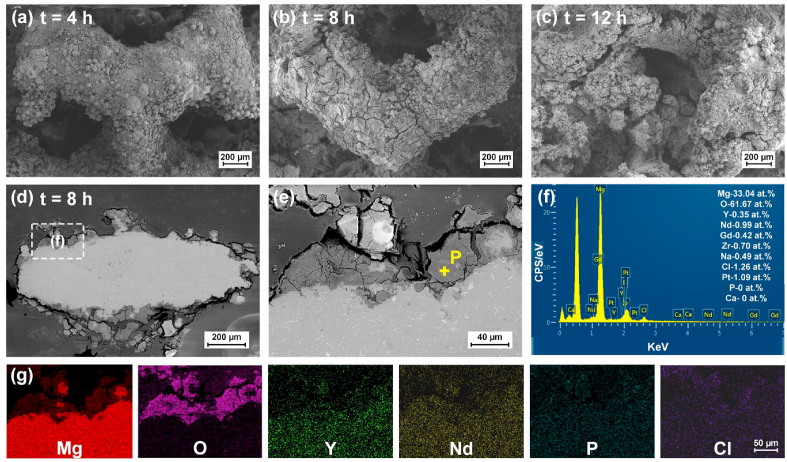
*In vitro* corrosion behavior (a–c) local surface after different periods, (d–e) cross sections, (f) EDS point analysis and (e) elemental mapping of corrosion products after 8 h.

According to Fig. 9(e), the corrosion products at the surface of scaffolds were composed of α-Mg, Y_2_O_3_ and Mg(OH)_2_. The MgRE precipitation phases were replaced by Mg(OH)_2_ compared with the substrate. The detailed morphology of corrosion products is shown in Fig. 10(a–c). After 4 h immersion, the corrosion products appeared continuous and covered the surface of scaffolds. The attached powder particles indicated that the corrosion products were not dense. When the immersion time reached 8 h, pronounced cracks were found. After 12 h immersion, the shapes of struts were difficult to be recognized due to severe corrosion and exfoliation. The cross sections of struts after 8 h immersion are further observed in Fig. 10(d–g). The corrosion products loosely attached to the surface of struts. Massive perforative cracks were found, indicating the lack of passivation effect. The EDS analysis in Fig. 10(f) reveals the composition at point P in the corrosion layer. The degradation products mainly contained Mg and O with the ratio of 1:2, implying the formation of Mg(OH)_2_.

**Fig. 11:**
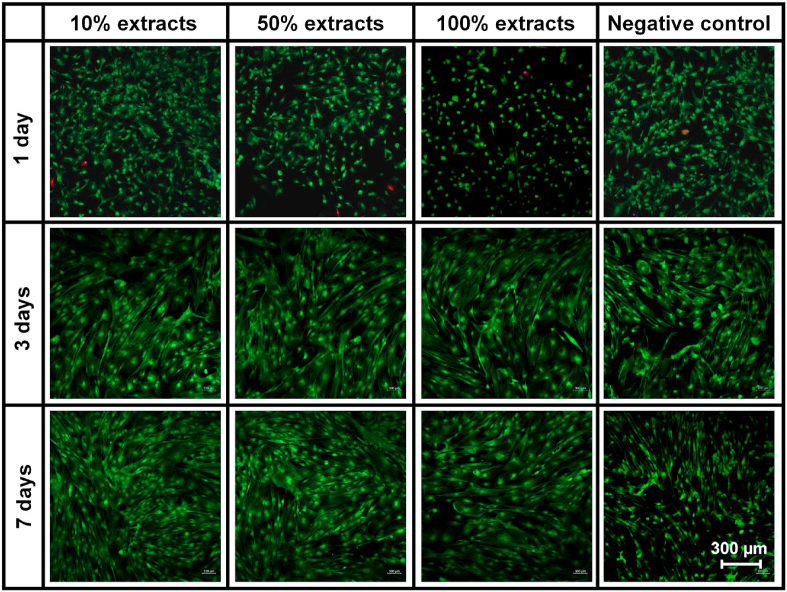
The living/dead staining results of BMSC cells cultured in extracts of different concentrations after different periods (green for living cells, red for dead cells). (For interpretation of the references to color in this figure legend, the reader is referred to the Web version of this article.)

**Fig. 12:**
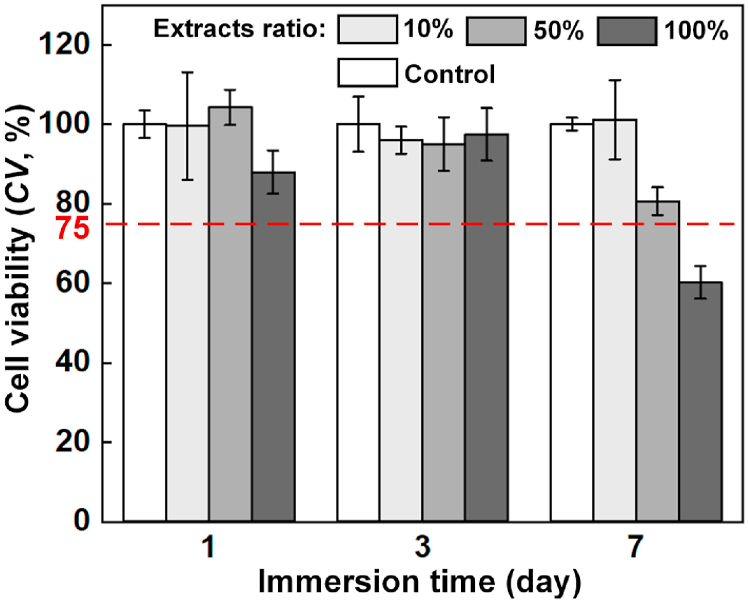
Cell viability of BMSC cells cultured in extracts of different concentrations after different periods.

Fig. 11 shows the living/dead staining results of BMSC cells cultured in 10%, 50% and 100% extracts for 1, 3 and 7 days. In general, the cells are widely distributed and in a healthy fusiform shape. It suggests a favorable biocompatibility of WE43 alloy. However, with increasing the concentration of extracts or culturing periods, the number of living cells decreased, and the shape of living cells collapsed. To further quantitively examine the cytotoxicity, the cell viability of BMSC cells in extracts of different concentrations was measured as shown in Fig. 12. Except the group in 100% extract after 7 days, all the other groups exceeded the cytotoxicity threshold (≥75%), showing acceptable cytocompatibility. For the culturing periods of 1 and 3 days, no definite pattern was observed regarding the influence of extract concentration on cell viability. After 7 days, the cell viability obviously reduced with increasing the extract concentration. Although the BMSC cells exhibited considerable endurance to the extracts of WE43 scaffolds, the enrichment of metal ions increased the cytotoxicity.

### 3.5 *In vivo* biodegradation, biocompatibility and osteogenic effect

**Fig. 13:**
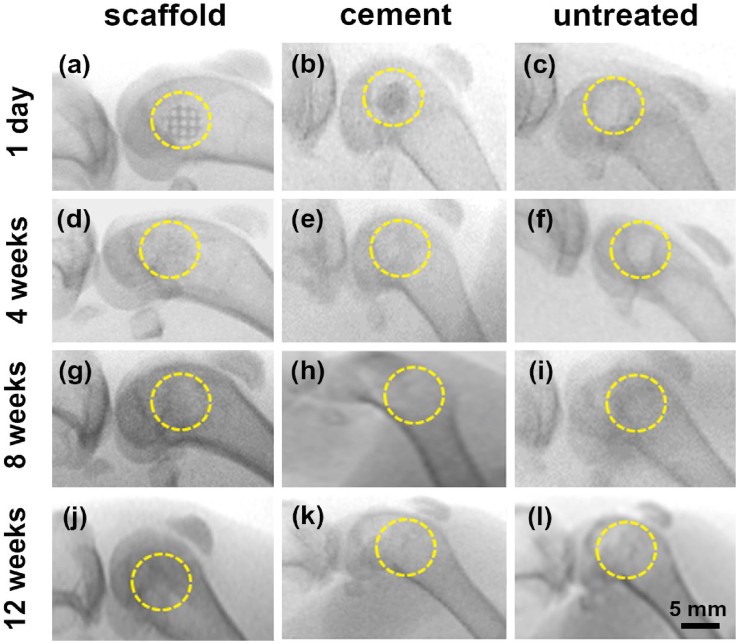
The postoperative serial X-rays of WE43 scaffolds, cement and untreated groups at different time points after the surgery. The yellow circles indicated the regions of preset femoral condyle defect. (For interpretation of the references to color in this figure legend, the reader is referred to the Web version of this article.)

All the rabbits appeared normal with similar living activities during the test. At each harvest timing, no signs of obvious evidences of inflammation, rejection reaction or infection were found by means of gross observation for all the samples, generally indicating good biocompatibility. As shown in Fig. 13(a–c), the X-rays at 1 day after the surgery revealed that the experimental models were successfully prepared. Complete structures were confirmed. At 4 weeks after the surgery, the structure of WE43 porous scaffold became indistinct and the residual broken pieces were observed (Fig. 13(d)), indicating a great amount of degradation and the collapse of structural integrity. In the cement group (Fig. 13(e)), the calcium sulfate cement had faded away without the sign of any residue. At 8 and 12 weeks after the surgery in Fig. 13(g–l), the serial X-rays showed gradual new bone regeneration occurred in all the three groups, evidenced by the high-density new tissues growing inside the defects.

**Fig. 14:**
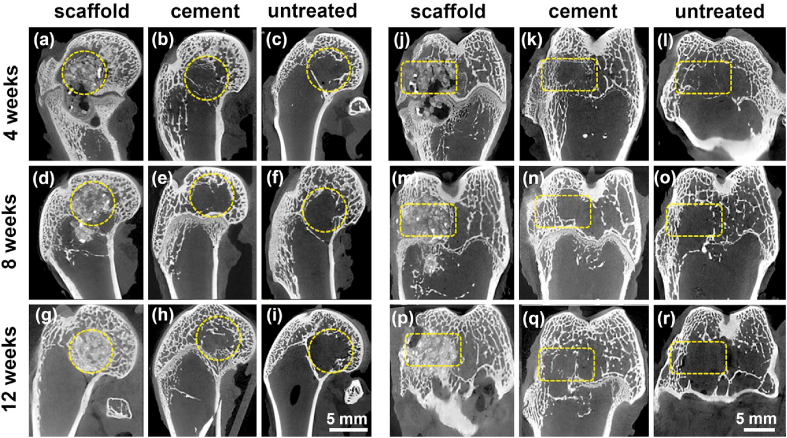
The postoperative sagittal and coronal Micro-CT scans of different groups respectively at different time points after the surgery. The yellow circles and boxes indicated the regions of the preset femoral condyle defect. (For interpretation of the references to color in this figure legend, the reader is referred to the Web version of this article.)

**Fig. 15:**
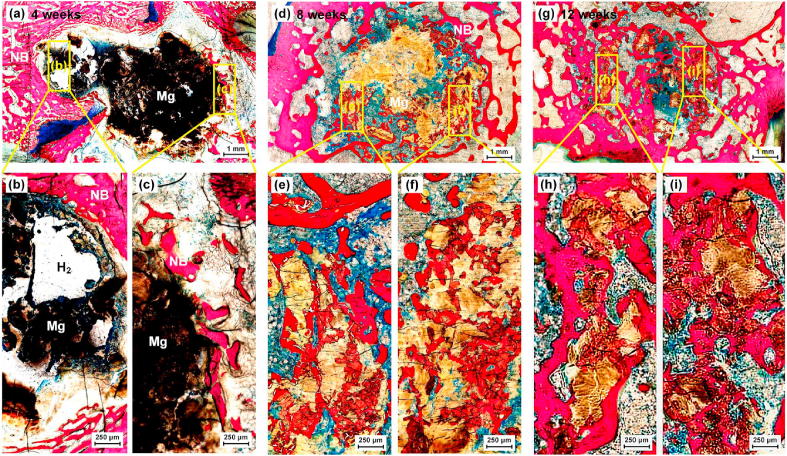
Methylene blue/acid fuchsin staining of the scaffold group at 4, 8 and 12 weeks (NB: newly-regenerated bone; Mg: WE43 alloy), samples were taken from the defect region. (For interpretation of the references to color in this figure legend, the reader is referred to the Web version of this article.)

Micro-CT scans were performed to further characterize the degradation of the implants and the progress of new bone regeneration as shown in Fig. 14. At 4 weeks after the surgery, a large part of WE43 scaffolds collapsed due to degradation (Fig. 14, Fig. 15j), losing the capacity of mechanical supporting. The clear defect boundary, shown in black color, existed with a small amount of regenerated bone trabeculae, shown in white color. Meanwhile, large gas bubbles were observed around the scaffold, and diffused into the marrow cavity. They were hydrogen according to the corrosion of Mg. Based on the CT images, the volume of bubbles was estimated 32.1 ± 15.9 mm^3^ in the distal femur region. In the cement group (Fig. 14(b, k)), the implanted cylinder had completely degraded, and no residue was visually observed. A considerable amount of new bone trabeculae had grown into the defect, but most of the defect area was still empty. With regard to the untreated group (Fig. 14(c, l)), only a tiny amount of new bone trabecular growth was observed, and the empty cavity inside the defect was significantly larger than that in cement group.

At 8 weeks after the surgery, the implanted WE43 scaffolds continued the degradation, and the original porous structure couldn't be recognized at all (Fig. 14(d, m)). However, no obvious accumulation of hydrogen gas was observed in the distal femur. The volume of hydrogen gas remaining in the distal femur region reduced to 1.73 ± 1.38 mm^3^, approximately 5% of the gas volume at 4 weeks, implying that the released gas bubbles disappeared with time. More trabecular bone regeneration was observed around the defect cavity, and some new bone partially formed visual connection with the broken WE43 structure. In the cement group (Fig. 14(e, n)) and the untreated group (Fig. 14(f, o)), more newly-regenerated trabecula was also observed in the defect area compared with that at 4 weeks, though the most area of the defects still kept empty.

At 12 weeks after the surgery, there were fewer WE43 residues in the defect region (Fig. 14(g, p)). Both the cavity and the scaffold were replaced with the visually white stuff, which appeared denser than that at 4 and 8 weeks, meanwhile the clear boundary of defect disappeared, both indicating that new bone had gradually grown inside the defect region as the scaffold further degraded. Besides, the accumulation of hydrogen gas was not evident anymore. In the cement group (Fig. 14(h, q)), more trabecular bone distributed in the defect region compared with that at 4 and 8 weeks. The trabecular structure was disordered and most of the cavity visually stayed empty however. For the untreated group (Fig. 14(i, r)), the defect area remained almost empty, and little bone growth was observed.

To further characterize the osteogenic effect induced by WE43 scaffolds after the surgery, hard tissue sections at the defect was performed. At 4 weeks after the surgery in Fig. 15(a–c), hydrogen gas bubbles were observed around the scaffold as marked by the white region, which matched the Micro-CT results. In addition, newly-regenerated bone, indicated as NB, was found to cling to the WE43 scaffold, indicated as Mg. The residual pieces of scaffolds were quite obvious. After 8 weeks as shown in Fig. 15(d–f), the majority of WE43 scaffold disappeared obviously, and the newly-regenerated bone spread in the crevices. Big hydrogen bubbles couldn't be observed. After 12 weeks in Fig. 15(g–i), the scaffold had degraded mostly and only scattered debris was observed. The newly-regenerated trabecular bone further filled in the defect zone. Overall, the promoted osteogenic effect was confirmed by the implanted WE43 porous scaffolds.

**Fig. 16:**
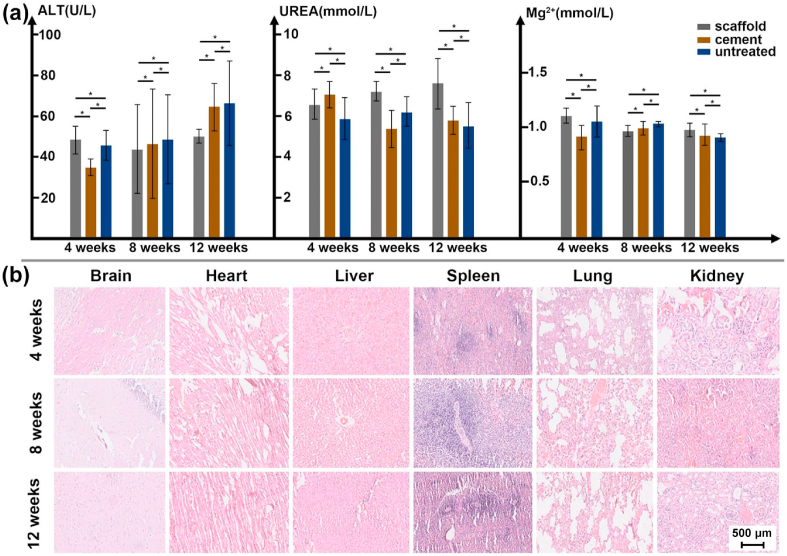
*In vivo* biocompatibility: (a) blood test indexes including ALT, UREA and Mg^2+^ concentration, (b) the HE staining of vital organs at different time points for the group of WE43 scaffolds, the same scale bar was used for different organs.

The *in vivo* biocompatibility was investigated by blood biochemistry and vital organs sectioning. As shown in Fig. 16(a), an obvious change or pattern was not observed regarding the values of ALT, UREA and Mg^2+^ concentration among different groups within implantation time. A slight increase of the ALT was detected with increasing the implantation time for the cement and untreated group. A slight increase of the UREA was observed for the WE43 scaffold group. Moreover, the concentration of Mg^2+^ in blood was not significantly influenced by the degradation of WE43 scaffolds. All the change of values with different time and groups fell within a normal range. It indicated that the degradation products of the fabricated WE43 scaffold did not cause significant damage effects on liver and kidney function. The continuous postoperative blood biochemistry test verified *in vivo* nontoxicity and good histocompatibility of the WE43 scaffolds.

Fig. 16(b) showed the HE staining results of vital organs, including brain, heart, liver, spleen, lung and kidney, at various time points. From 4 weeks to 12 weeks after the surgery, the HE staining could clearly show the basic structure units including nerve cells and gliocytes (brain), myocardial fibers and cells (heart), hepatic lobules and hepatocytes (liver), splenic pulp (spleen), pulmonary lobules and bronchioles (lung), and glomerulus and tubules (kidney). No sign of tissue damage was detected, such as congestion or ischemia, structural disorder, and inflammatory cell infiltration, etc. No pathological degeneration, necrosis or apoptosis occurred. In general, the degradation of WE43 scaffolds didn't cause damage to the vital organs.

## 4 Discussion

### 4.1 Processing optimization and formation quality

As Table 1 shows, Mg has unique properties compared with commonly used metals such as Fe and Ti. For comparison, the properties of Y were also listed. They have huge influence on the L-PBF process of WE43 alloy. The high susceptibility to oxidation and vaporization of Mg requires a special consideration on the laser energy input and shielding gas flow during the L-PBF of WE43 porous scaffolds. The oxidability of Y, Nd and Gd in WE43 is even higher than that of Mg. The natural passivation caused by oxidation is unavoidable during the operation of WE43 powder considering the high specific area of powder particles. The oxide shells at the surface of WE43 powder were observed as shown in Fig. 1(c), which was beneficial to prevent possible fire accidents during the powder operation, considering the good passivation effect of Y_2_O_3_ with the R_PB_ value as 1.13. During the L-PBF, good shielding atmosphere is essential to prevent melting from new oxidation, meanwhile a high energy intensity is necessary to break the oxide shells to avoid big oxide inclusions. The used laser spot was 70 μm in diameter, generating an energy intensity about 1.6 × 10^6^ W/cm^2^ when the laser power was 60 W. As shown in Figs. 6 and 7, the oxides existed in form of scattered flakes in size of 1–10 μm after the L-PBF. The high energy intensity caused massive vaporization during the L-PBF. The vaporization fume and spatter, which could attenuate the laser energy absorption on powder bed, were eliminated by the circular gas system to achieve stale melting as shown in Fig. 2(b). The recoil force of vaporization formed a keyhole inside the molten pool, and the porosity is directly related to the laser energy input [54]. Excessive laser energy input resulted to severe vaporization and its by-products such as fume, spatter and porosity. A small energy input was favorable as much as it could avoid lack of fusion for the L-PBF of Mg alloys, explaining that good fusion quality (*ρ*_*S*_>99.5%) couldn't be achieved when *P*_*L*_ was over 90 W or *V*_*S*_ over 900 mm/s as shown in Figs. 3 and 4. Moreover, Mg has a high thermal expansion coefficient, a relatively small energy input as well as preheating were significant to inhibit thermal stress and distortion. Overall, the L-PBF processing window of WE 43 alloy was much narrower than that of Ti and Fe alloys.

**Table 1:** Material properties of pure metals Mg, Y Ti and Fe.

Properties	Unit	Value			
		Mg	Y	Ti	Fe
Density (T_1_)	g/cm^3^	1.74	4.47	4.51	7.874
Melting point	°C	650	1526	1668	1538
Boiling point	°C	1091	2930	3287	2862
Heat conductivity (T_1_)	W/m∙K	158	17.2	21.9	80
Thermal expansion (T_1_)	10^−6^/K	24.8	10.6	8.6	11.8
Surface tension (T_m_)	mN/m	559	804	1590	1835
Viscosity (T_m_)	mPa∙s	1.25	3.97	4.0	6.93
Standard electrode potential (T_1_)	V	−2.37	−2.37	−1.63	−0.45
R_PB_	/	0.81	1.13	1.76	2.14

T_1_: 20 °C, T_m_: melting point.

Good fusion quality was achieved with the optimized laser energy input (*P*_*L*_ = 60 W and *V*_*S*_ = 600 mm/s). However, powder attachment at the surface resulted to a thicker strut than the designed one, and deteriorated the dimensional accuracy of porous scaffolds as shown in Fig. 4. As shown in Table 1, the density, melting point, surface tension and the viscosity of Mg are much lower than those of Ti and Fe. During laser melting, plenty of molten liquid and powder particles are ejected out from the molten pool due to vaporization. They fall down and transform into spherical balls due to surface tension. The ejection also disturbs the adjacent powders, and pushes them away from the molten pool. The solidified spatters and partially melted powders cause thicker struts. The thicker strut is also greatly resulted from the horizontal movement of liquid metal. The powder bed is in a stochastic stacking configuration and has a very low thermal conductivity. Powder particles away from the molten pool are also possibly to melt due to the heat accumulation and wetting. Liquid metal sucks into the surrounding powder and results to powder attachment. The geometrical error between the designed and fabricated porous scaffolds can result to a huge variation of properties.

The optimized laser energy input is a bit lower for porous scaffolds compared with that for bulk samples, explained by the effect of fusion volume and heat accumulation. Moreover, fusion quality is the first priority for bulk samples, indicating that only the optimized laser energy input is necessary for most occasions; while fusion quality and geometrical accuracy have the equal importance for properties of porous scaffolds. The optimized LPBF processing conditions for bulk samples may not work for porous scaffolds. However, the influence of porous design and manufacturing process on the properties and applications has been neglected to a great extent regarding biodegradable Mg alloy porous scaffolds [29]. The current status is majorly contributed to the interdisciplinary considerations. Mechanical experts concern themselves with design and manufacturing; material professionals pay more attention on alloying design and properties characterization; while medical doctors are interested in therapeutic effects. Even the porous design is optimized, the discrepancy of formation quality definitely becomes a critical issue to the performance evaluation. By using the CES, both good fusion quality and low dimensional error were achieved with the customized *P*_*L*_, *V*_*S*_ and *φ*_*c*_. The *P*_*L*_ and *V*_*S*_ were mainly decided by the powder characteristics, while the *φ*_*c*_ was adjusted according to the geometrical features and the optimized laser energy input.

### 4.2 Microstructure and mechanical properties

The high strength of WE43 alloy is primarily attributed to solid solution, refined grains and precipitated phases [12]. The lattice parameters of RE elements are similar to those of Mg. The solid solubility of Y is as high as 12.5 wt% in the Mg substrate. The addition of RE and Zr elements not only increases the nucleation particles, but also inhibits the grain growth, thus resulting to refined grains. Various MgRE precipitation phases form during the solidification and cooling of WE43 alloy. The size and distribution of precipitation phases play a significant role to the mechanical properties of WE43. The moving laser beam and additive thermal cycles during L-PBF generate a rapid cooling rate, a big thermal gradient and overlapped heat affected zones, which decides the microstructures of WE43 porous scaffolds together with the addition of alloying elements.

As shown in Figs. 6 and 7, the microstructure of WE43 porous scaffolds were composed of α-Mg, Y_2_O_3_ and MgRE precipitates, consistent with the previous literatures [[43], [44], [45], [46], [47], [48], [49], [50], [51], [52]]. This work firstly reported the influence of strut size on the microstructure of porous scaffolds. A bigger strut size results to more heat accumulation and a slower cooling rate. The grain size of α-Mg and the amount of MgRE precipitates increases with increasing strut sizes under the same energy input. The solute RE atoms in α-Mg decreases with increasing strut sizes correspondingly. It should be mentioned that the volume ratio of oxides keeps the similar for different strut sizes, since they originate from the starting powder. The MgRE precipitated phases mainly include intergranular Mg_14_(Nd,Gd)_2_Y and intragranular Mg_41_RE_5_. The primary Mg_14_(Nd,Gd)_2_Y crystalizes directly from the molten pool due to the constitutional supercooling. Since the cooling rate is extremely fast at the solidification front during the L-PBF, very fine α-Mg grains with extra solution of RE elements precipitate from the molten pool. Mg_41_RE_5_ precipitates in form of nano-sized short needles inside the α-Mg grains during the subsequent multiple thermal cycles during the L-PBF. Meanwhile, Mg_14_(Nd,Gd)_2_Y grow larger into dispersed globular particles along the grain boundaries owing to the repeated thermal cycles.

As Fig. 8(a) shows, the hardness slightly decreased with increasing the strut size, possibly explained by the increased grain size. According to the Gibson-Ashby law, the strength and stiffness of porous scaffolds decreases exponentially with increasing the structural porosity [29]. With increasing the strut size, the structural porosity decreases. For diamond scaffolds in Fig. 8(b), the *CS* and *YM* increased exponentially with increasing strut sizes. The *CS* and *YM* of S500D scaffolds (DP = 72.7%, FP = 70.8%) were 4.9 and 5.1 times those of S300D scaffolds (*DP* = 89.6%, FP = 88.6%) respectively. A 1.2-fold increase in porosity approximately resulted to a 5-fold decrease in loading capacity namely. The dimensional error causes a large change of porosity between design and fabrication. The reduced geometrical error by the CES gives a better control of mechanical properties therefore. To further diminish the geometrical error and improve surface roughness of porous scaffolds, post treatments such as acid and electrolysis polishing are recommended [[50], [51], [52], [53]]. With the similar structural porosity, the mechanical properties of porous scaffolds also depend on the type of porous units as shown in Fig. 8(e–f). BCC scaffolds showed the highest compressive strength, since they have vertical struts in parallel with the compression direction. Diamond scaffolds exhibited the lowest compressive strength, since they have interconnected joints working as the weak points during the compression. Lattice gyroid scaffolds have arc shaped struts and smooth transition, so their strength was higher than that of diamond scaffolds. Sheet gyroid scaffolds exhibited higher strength than that of lattice gyroid ones owing to their continuous loading characteristics, but their permeability is regarded not good for bone implants [21,29,55]. Additively manufactured WE43 porous scaffolds provide huge potential to adjust the mechanical performance of bone implants by adjusting pore units in shape, size and distribution [66], however, the controlling of formation quality, including good fusion quality and high dimensional accuracy, is the precondition to realize the design purpose.

### 4.3 Biodegradation, biocompatibility and osteogenic effect

The *in vitro* immersion test revealed an excessively fast degradation rate for the as-built WE43 porous scaffolds, and the scaffolds collapsed into small pieces just after 16 h immersion. The pH value and the generated volume of hydrogen increased with the degradation. Mg has a considerably low standard electrode potential as −2.37V, and the R_PB_ value of MgO is 0.81, both indicating that Mg is highly reactive in aqueous environment. The corrosion layers contained α-Mg, Y_2_O_3_ and Mg(OH)_2_. The fast degradation rate was explained by the massive secondary phases and the high specific area of the porous scaffolds. The secondary phases promoted the degradation by forming galvanic reaction with the α-Mg substrate; while the high specific area, resulted from the rough surface and the interconnected pores, increased the degradation by the enlarged contact surface with the corrosive fluid. The observed degradation rate is much faster than some literatures [50,51]. There are many factors greatly influencing the corrosion behavior of the same material observed by different *in vitro* immersion tests, such as immersion fluid, specimen size and geometry, composition and microstructure. It commonly acknowledges that the test conditions from different groups may give quite different results even regarding the same materials or structures. The difference in L-PBF processing conditions, surface morphology and structural geometry may also help to explain the faster degradation rate compared with the literatures. Despite the fast degradation, the living/dead staining results of BMSC cells cultured in 10%, 50% and 100% extracts for 1, 3 and 7 days generally indicated acceptable cytotoxicity. The cell viability decreased with increasing the extraction ration, explained by the enrichment of metal ions.

According to the *in vivo* investigation, the WE43 scaffolds lost their structural integrity at 4 weeks after the surgery. The bone healing time varies for different fracture sites, and the mechanical support provided by implants should be sustained for 12–24 weeks depending on the clinical conditions [6]. Clinical trials reported the complete degradation of Mg alloy screws at 6 and 12 months [3,4], which seems to meet the degradation requirement. The *in vivo* degradation rate of the used WE43 porous scaffolds are regarded too fast to match the bone reconstruction. Degradation of WE43 porous scaffolds enables a series of reactions within the physiological environment leading to the formation of hydrogen gas and metal ions. Theoretically, a milligram of pure Mg generates hydrogen gas in volume of 1.06 ml. The implanted WE43 scaffold weighs approximately 50 mg, so the total degradation roughly release 50 ml hydrogen gas. Excessive hydrogen gas can interfere with the bone healing process, resulting in callus formation and cortical defects [34].The gas bubbles were evident at 4 weeks after the surgery, indicating massive degradation. After 8 and 12 weeks, the gas bubbles almost visually disappeared. The absence of hydrogen gas on one hand indicates that the majority of WE43 scaffolds degraded in the first 4 weeks, on the other hand implies that the generated hydrogen gas can be absorbed, transported and metabolized in the organism. Therefore, if the hydrogen gas is generated in a controlled rate, the resulting negative effect can be inhibited. In the future, extra measures, such as surface treatment and heat treatment [[8], [9], [10],^48^,^68^], are regarded necessary to control the degradation rate of the WE43 porous scaffolds fabricated by L-PBF.

The favorable osteoinduction and osseointegration resulted by the biodegradation of Mg alloy implants have been widely reported by literatures [[1], [2], [3],[13], [14], [15],^69^]. As shown in Fig. 14, accompanied with the gradual degradation of WE43 scaffold, new trabecular bone grew around in the early stage, and then increasingly grew inside the degraded scaffold. At 12 weeks after the surgery, the bone defect had been filled with a large amount of new trabecula. The histological staining further confirmed that the residual debris of WE43 scaffold and new trabecular bone formed a tight complex, providing sufficient mechanical support for the surrounding skeletal structure (Fig. 15). While as for the bone regeneration progress in cement and untreated groups, even at 12 weeks postoperatively, a wide range of empty cavity still existed in the bone defect. This failure of bone repair will cause the surrounding bone and articular surface to lose effective mechanical support, and fractures and articular collapse are prone to happen when joint moves.

Considering the interaction between the degradation and the body, the biocompatibility of WE43 scaffolds is the fundamental issue. Mg is an essential element on bone metabolism, its contents in the human body and threshold values for daily intake are both considerably tolerant. For example, the inhibiting concentration (IC_50_) of Mg^2+^ ion to osteoblast cells was estimated greater than 4.02 mmol/L, and the IC_50_ to endothelial cells roughly 66.7 mmol/L. The median lethal dose (LD_50_) was approximately 5000 mg/kg [7]. The total mass of the used WE43 scaffold was approximately 50 mg, much lower than the threshold values even considering the total degradation in a short time. For comparison, the Magnezix 3.2 mm compression screw, which was based on WE43 alloy and achieved CE mark in 2013, weighted roughly 150 mg for a length of 10 mm [70]. With the structural porosity, the used volume of WE43 scaffold can be reduced as long as sufficient mechanical supporting is provided to treat the bone defects.

Compared with the widely reported Mg, the mechanism and effect of RE elements on the biocompatibility have been insufficiently studied. RE elements in WE43, including Y, Nd and Gd, naturally don't exist in biochemical processes. However, it was reported that they can interact with a large number of biological pathways, greatly due to their close similarity in the ionic radius to that of the massive life element Ca^2+^ ion [11]. Since the free RE ions tends to bind with various constituents of physiological fluids, it is difficult of make the quantitative evaluation on their toxic effect unless the RE compound can be accurately detected. The LD_50_ of Y, Nd, and Gd by the injection of mice are 88, 600 and 550 mg/kg in form of chloride salts, while ranged 181–1759 mg/kg for Y in form of nitrate for example [11,71]. Moreover, similar mechanism, along with biological transport pathways, can result to varied toxicity due to exposure routes. Although the impact of individual RE elements in isolation shows some potential concerns [11,12], the overall physiological response to the use of complete alloys suggests these concerns are not insurmountable, and the long-term impact of RE elements on the biocompatibility needs further investigation.

## 5 Conclusions

This work investigated the influence of L-PBF energy input and scanning strategy on the formation quality of WE43 porous scaffolds, and characterized the microstructure, mechanical properties, biocompatibility, biodegradation and osteogenic effect of the as-built WE43 porous scaffolds by *in vitro* and *in vivo* investigations.

WE43 porous scaffolds of good formation quality were manufactured by L-PBF with the customized lase energy input and scanning strategy (CES). The proposed CES includes the optimization of laser power, scanning speed and offset spacing. The relative density of struts reached over 99.5%, and the fabrication error of structural porosity reduced less than 10%. The microstructure of WE43 porous scaffolds was composed of α-Mg, Y_2_O_3_ and MgRE precipitate. The compressive strength and Young modulus ranged 4.37–23.49 and 154.40–873.02 MPa, dependent on the porous design. The existence of massive secondary phases as well as the enlarged specific surface area of WE43 porous scaffolds resulted to an excessively rapid degradation rate. The structural integrity was seriously damaged after 12 h by immersion test in Hanks solution and after 4 weeks by implantation test in the rabbits’ femur. Good biocompatibility was observed by *in vitro* cell viability, *in vivo* blood test and HE staining results of vital organs. Promoted osteogenic effect was confirmed after 8 and 12 weeks implantation of WE43 scaffolds compared with those of cement and untreated groups. In the future, the degradation rate needs to be controlled to provide sufficient mechanical support during bone healing.

## CRediT authorship contribution statement

**Jinge Liu:** Conceptualization, Investigation, Data curation, Writing – original draft. **Bingchun Liu:** Investigation, Writing – original draft, Data curation. **Shuyuan Min:** Investigation, Data curation. **Bangzhao Yin:** Investigation. **Bo Peng:** Software. **Zishi Yu:** Investigation. **Caimei Wang:** Resources. **Xiaolin Ma:** Investigation. **Peng Wen:** Supervision, Resources, Writing – review & editing. **Yun Tian:** Supervision, Resources, Writing – review & editing.

## Declaration of interests

The authors declare that they have no known competing financial interests or personal relationships that could have appeared to influence the work reported in this paper.
