# Supplementary material for: Biodegradable magnesium alloy WE43 porous scaffolds fabricated by laser powder bed fusion for orthopedic applications: Process optimization, in vitro and in vivo investigation
Source: Bioact Mater. 2022 Feb 24;16:301–19. doi: 10.1016/j.bioactmat.2022.02.020 (PMC8965912; doi:10.1016/j.bioactmat.2022.02.020)
Supplement: Multimedia component 1 [file mmc1.docx]

**1. Surgical details of *in-vivo* test**

At one day before surgery, all rabbits got food and water removed to relieve the potential gastrointestinal reaction during surgical procedure. General anesthesia was conducted via the ketamine hydrochloride (50 mg/kg, intravenous injection) and fentanyl (0.17 mg/kg, intramuscular injection). After being positioned appropriately, rabbits’ left knees were shaved carefully and cleaned with iodophor for three times. The femoral condyle was exposed via routine lateral parapatellar approach. Then we applied electric drill to create the lateral condyle cylindrical defects. The surgical region was continuously flushed with sterile saline solution to minimize heat generated from drilling process. Depending on different experiment groups, the defects were left empty or repaired respectively by filling WE43 scaffolds or cement columns (Fig. 1s). The composition of the bone cement was calcium sulfate hemihydrate (Wright Medical Technology, USA). After confirming the stability of implants, overlying muscles and skin were closed in layers. Postoperatively, all rabbits were housed in separated cages and allowed to move without restriction. In addition, intramuscular antibiotics (cefazolin sodium, 0.2 g/kg) were administered to prevent local infection in the early five days. During the follow-up, we observed rabbits’ general conditions with time, including dietary and mental status, knee range of motion, walking gait, and wound recovery. At 4, 8 and 12 weeks after surgery, rabbits were respectively sacrificed by means of euthanasia and their distal femur samples were collected for the following experiment steps. Five samples were harvested at each timing in each group, and timely replacement would be finished once unexpected failure or death occurred.


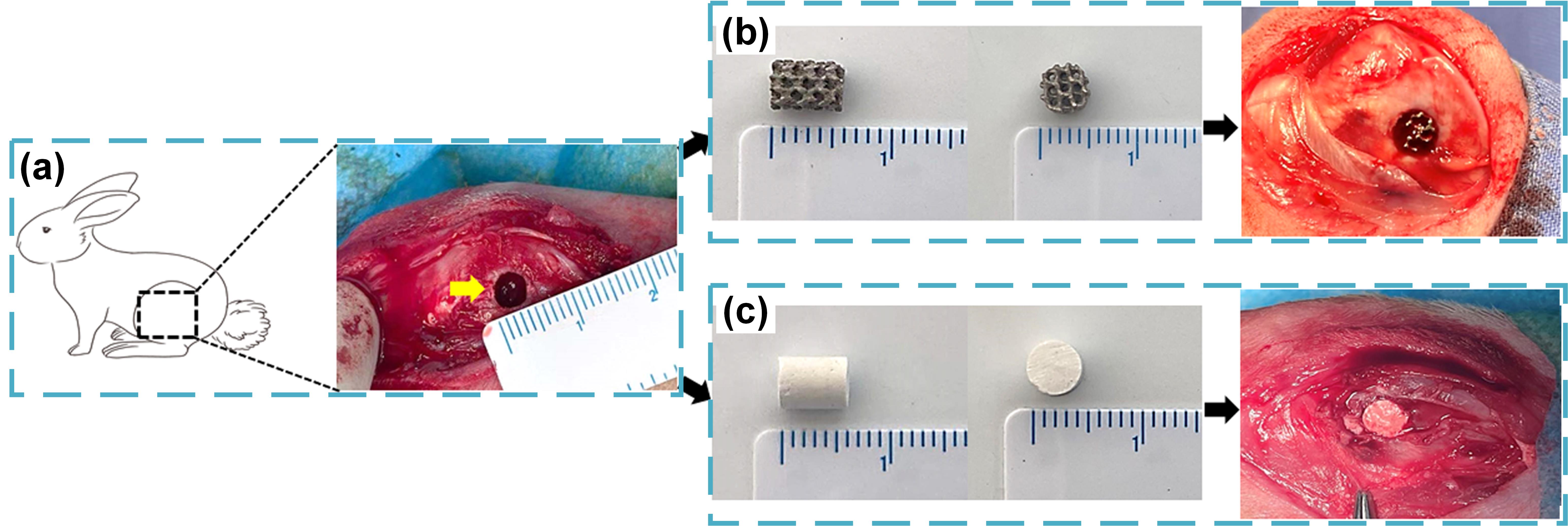


Figure S1. The flow graph for the repair of femoral condyle defect: (a) the lateral condyle cylindrical defects; (b) the repair process of scaffold group;(c) the repair process of cement group.

**2. Pictures of S400D and S500D porous scaffolds**

As Fig.S2 and S3 show, the struts get thicker with increasing the heat input (*P_L_*/*V_S_*). Namely, the fabricated structural porosity decreases with increasing the heat input though the designed structural porosity is the same. The increased heat input results to a larger molten pool, a higher temperature and a slower cooling rate, which increases the attachment of powder particles. The decreased heat input alleviates the attachment of powder particles, resulting to a better fabrication accuracy, but deteriorates the fusion quality due to insufficient melting of powder particles.


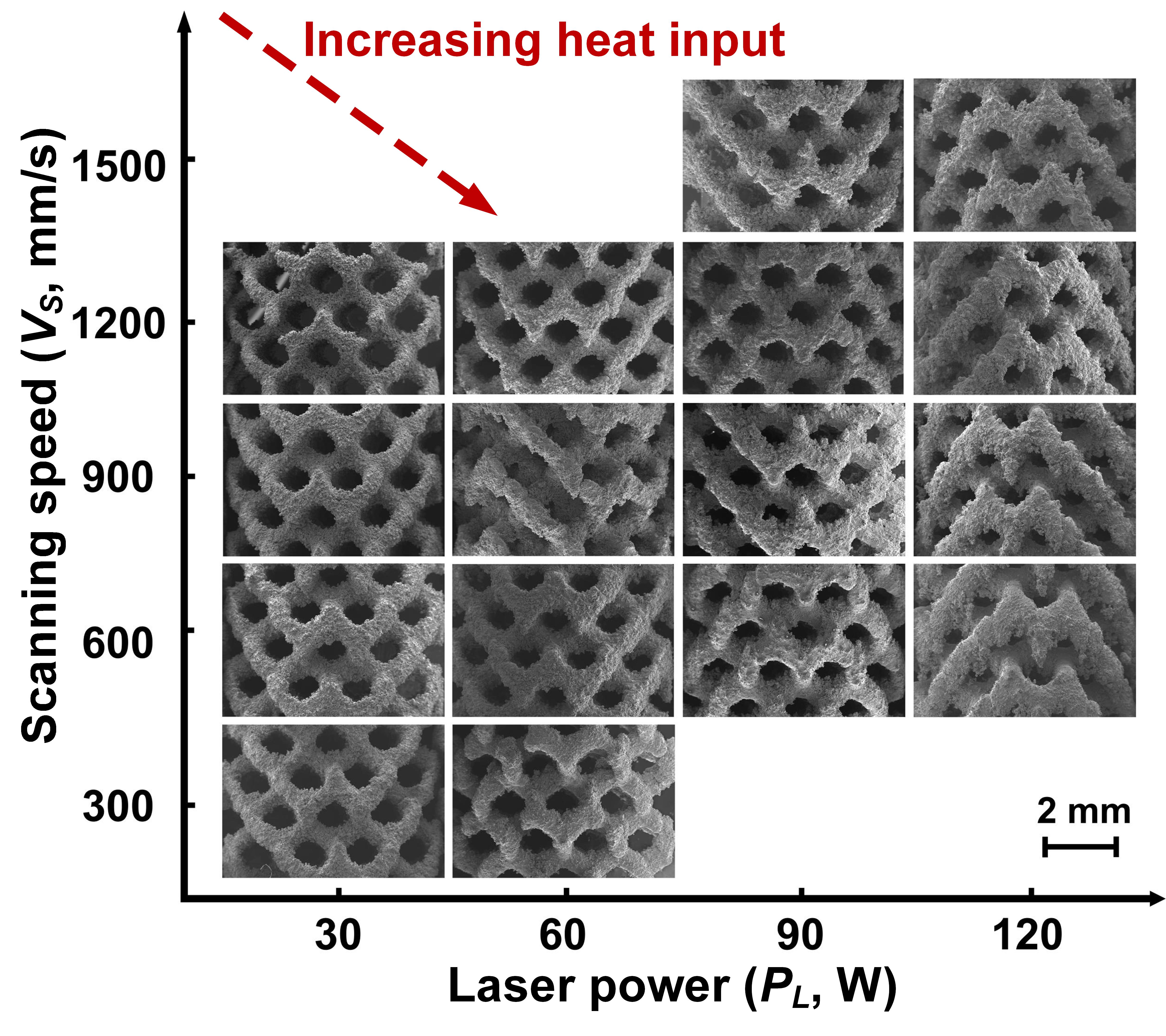


Figure S2. Pictures of S400D porous scaffolds with various laser energy input.


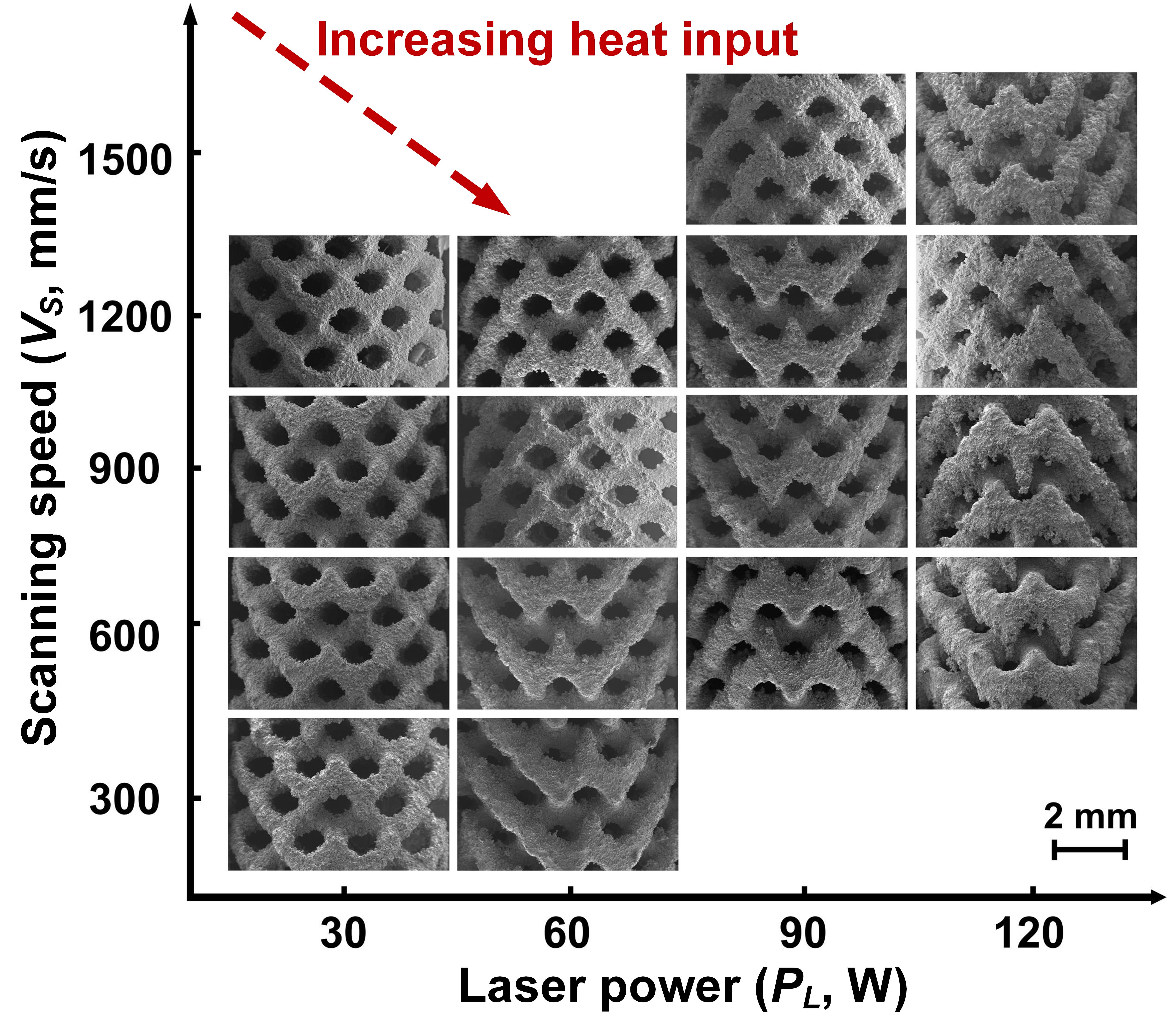


Figure S3. Pictures of S500D porous scaffolds with various laser energy input.

**3. The EDS point analysis results in Fig.9**

The energy dispersive X-ray spectroscopy (EDS) was applied to analyze chemical compositions of interested points in Fig.9. The chemical compositions at P1 point indicate the α-Mg substrate. The elemental distribution at P2 point indicate the formation Y_2_O_3_ with 63 at.% Mg, 16.57 at.% Y, 17.52 at.% O. In addition, a few Zr rich region mixed up with the Y_2_O_3_, which was confirmed as (Y, Zr)_2_O_3_ with the elemental composition (P3) of 62.08 at.% Mg, 16.58 at.% Y, 15.56 at.% O, 4.16 at.% Zr. The elemental result at P4 point is 85.97 at.% Mg, 4.84 at.% Y, 6.19 at.% Nd and 1.33 at.% Gd. At P5 point, 85.38 at.% Mg, 4.87 at.% Y, 6.43 at.% Nd and 1.24 at.% Gd are detected. Primary Mg_14_(Nd,Gd)_2_Y phases are confirmed. The precipitation phases inside the grain in shape of dot dash line are confirmed as Mg_41_RE_5_. The corresponding elemental compositions (P6) are 93.58 at.% Mg and 5.33 at.% RE. The Zr segregation region (P7) is confirmed as the coprecipitation of Mg_41_RE_4_ and Zr with the content of 89.04 at.% Mg, 5.69 at.% RE and 4.08 at.% Zr.

Table S1. The chemical compositions at Points 1-7 in Fig. 9 (wt.%).

| Point | Mg | O | Y | Nd | Gd | Zr | Possible phases |
| --- | --- | --- | --- | --- | --- | --- | --- |
| 1 | 96.43 | 2.34 | 0.072 | 0.2 | 0.18 | 0.12 | α-Mg |
| 2 | 63 | 17.52 | 16.57 | 0.92 | 0.67 | 1.30 | Y_2_O_3_ |
| 3 | 62.08 | 15.56 | 16.58 | 0.92 | 0.7 | 4.15 | (Y,Zr)_2_O_3_ |
| 4 | 85.38 | 1.98 | 4.87 | 6.43 | 1.24 | 0.09 | Mg_14_Nd_2_Y |
| 5 | 85.97 | 1.52 | 4.84 | 6.19 | 1.33 | 0.16 | Mg_14_Nd_2_Y |
| 6 | 93.58 | 0.99 | 2.04 | 2.84 | 0.45 | 0.09 | Mg_41_RE_5_ |
| 7 | 89.04 | 1.14 | 2.84 | 2.30 | 0.59 | 4.08 | Mg_41_RE_5_+Zr |

**4. The fitted curve of customized offset spacing for porous scaffolds of different porous units**

The customized energy input and scanning strategy (CES) is attempted to fabricate porous scaffolds with different types of pore units including body centered cube (BCC, S=486 μm), lattice gyroid (LG, S=510 μm) and sheet gyroid (SG, S=262 μm). The strut size S is adjusted to make the structural porosity of different scaffolds as 80% as the same as that of diamond scaffolds with S=400 μm. The least squares method is used to fit the curve of *φ_c_* with different *S* by using the experimental data of S300D, S400D and S500D scaffolds, as shown in Fig.S4.


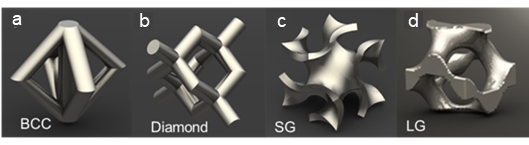


**e**


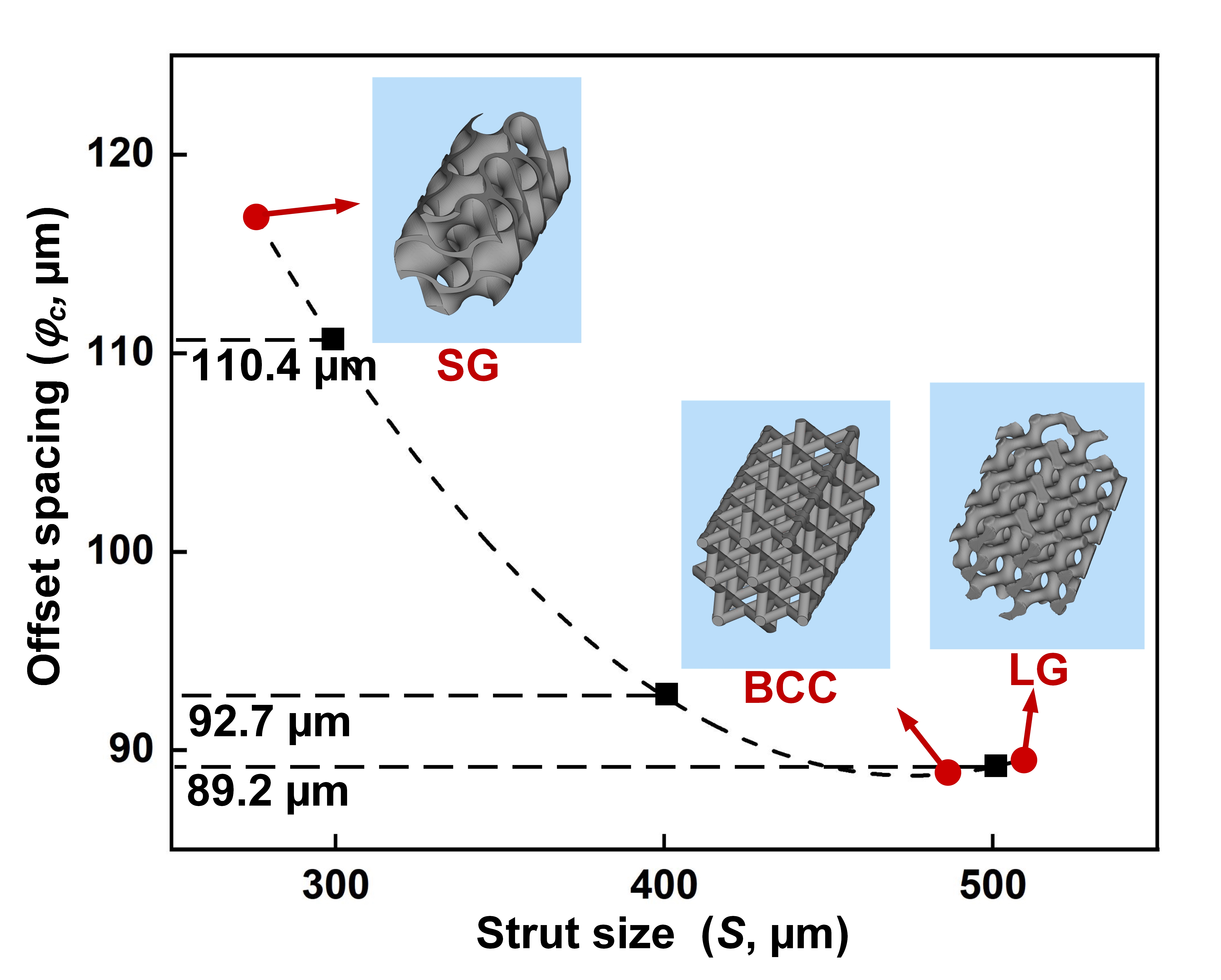


Figure S4. Different types of porous units a) body centered cubic BCC, b) diamond, c) sheet gyroid SG, d) lattice gyroid LG, and 3) a fitted curve of customized offset spacing *φ_c_* for different struct sizes
